# Dominant role of adult neurogenesis‐induced structural heterogeneities in driving plasticity heterogeneity in dentate gyrus granule cells

**DOI:** 10.1002/hipo.23422

**Published:** 2022-05-13

**Authors:** Sameera Shridhar, Poonam Mishra, Rishikesh Narayanan

**Affiliations:** ^1^ Cellular Neurophysiology Laboratory Molecular Biophysics Unit, Indian Institute of Science Bangalore Karnataka India

**Keywords:** adult neurogenesis, BCM rule, calcium signaling, degeneracy, dentate gyrus, heterogeneity, intrinsic excitability, synaptic plasticity, theta burst stimulation

## Abstract

Neurons and synapses manifest pronounced variability in the amount of plasticity induced by identical activity patterns. The mechanisms underlying such plasticity heterogeneity, which have been implicated in context‐specific resource allocation during encoding, have remained unexplored. Here, we employed a systematic physiologically constrained parametric search to identify the cellular mechanisms behind plasticity heterogeneity in dentate gyrus granule cells. We used heterogeneous model populations to ensure that our conclusions were not biased by parametric choices in a single hand‐tuned model. We found that each of intrinsic, synaptic, and structural heterogeneities independently yielded heterogeneities in synaptic plasticity profiles obtained with two different induction protocols. However, among the disparate forms of neural‐circuit heterogeneities, our analyses demonstrated the dominance of neurogenesis‐induced structural heterogeneities in driving plasticity heterogeneity in granule cells. We found that strong relationships between neuronal intrinsic excitability and plasticity emerged only when adult neurogenesis‐induced heterogeneities in neural structure were accounted for. Importantly, our analyses showed that it was not imperative that the manifestation of neural‐circuit heterogeneities must translate to heterogeneities in plasticity profiles. Specifically, despite the expression of heterogeneities in structural, synaptic, and intrinsic neuronal properties, similar plasticity profiles were attainable across all models through synergistic interactions among these heterogeneities. We assessed the parametric combinations required for the manifestation of such degeneracy in the expression of plasticity profiles. We found that immature cells showed physiological plasticity profiles despite receiving afferent inputs with weak synaptic strengths. Thus, the high intrinsic excitability of immature granule cells was sufficient to counterbalance their low excitatory drive in the expression of plasticity profile degeneracy. Together, our analyses demonstrate that disparate forms of neural‐circuit heterogeneities could mechanistically drive plasticity heterogeneity, but also caution against treating neural‐circuit heterogeneities as proxies for plasticity heterogeneity. Our study emphasizes the need for quantitatively characterizing the relationship between neural‐circuit and plasticity heterogeneities across brain regions.

## 1 INTRODUCTION

Neurons and synapses of the same subtype receiving identical plasticity‐inducing activity patterns do not manifest identical levels of plasticity. Instead, they exhibit *plasticity heterogeneity* across synapses and neurons, manifesting as pronounced variability in the observed changes. There are several lines of evidence from in vitro and in vivo electrophysiological experiments for such plasticity heterogeneity, spanning different neuronal and synaptic subtypes (Beck et al., 2000; Bliss & Lomo, 1973; Davis et al., 2004; Greenstein et al., 1988; Kobayashi et al., 2013; Koranda et al., 2008; Larson & Munkacsy, 2015; Li et al., 2017; McHugh et al., 2007; Pavlides et al., 1988; Rathour & Narayanan, 2019; Shors & Dryver, 1994; Sjostrom et al., 2008; Wang et al., 1997). Although such plasticity heterogeneity has typically been overlooked in analyzing the impact of plasticity protocols, a growing body of experimental evidence identifies crucial roles for plasticity heterogeneity in neural encoding and storage. Specifically, the ability of neurons and synapses to undergo differential plasticity is critical for *context‐specific recruitment/allocation of a subset of* neurons and synapses during encoding processes (Aimone et al., 2014; Dieni et al., 2013; Ge et al., 2007; Huckleberry & Shansky, 2021; Josselyn & Frankland, 2018; Josselyn & Tonegawa, 2020; Lau et al., 2020; Lodge & Bischofberger, 2019; Park et al., 2016; Pignatelli et al., 2019; Schmidt‐Hieber et al., 2004; Yiu et al., 2014). The lack of plasticity heterogeneity would result in a scenario where all neurons and synapses undergo similar amount of plasticity for any given context. Such a scenario would erase the possibility of sparse and context‐specific recruitment of neural resources. Despite these well‐recognized roles of plasticity heterogeneity in context‐specific resource allocation, the *mechanisms* underlying these heterogeneities have not been assessed. Furthermore, there are postulates and lines of evidence for heterogeneities in intrinsic excitability playing a role in determining selective resource allocation (Aimone et al., 2014; Dieni et al., 2013; Ge et al., 2007; Huckleberry & Shansky, 2021; Josselyn & Frankland, 2018; Josselyn & Tonegawa, 2020; Lau et al., 2020; Lodge & Bischofberger, 2019; Park et al., 2016; Pignatelli et al., 2019; Schmidt‐Hieber et al., 2004; Yiu et al., 2014). However, the quantitative link between such cellular‐scale heterogeneities and plasticity heterogeneity has not been systematically assessed.

Granule cells (GCs) in the dentate gyrus (DG) offer an efficient system for addressing questions on the cellular mechanisms underlying plasticity heterogeneity. First, the pronounced biophysical heterogeneities in these cell types have been electrophysiologically well‐characterized (Heigele et al., 2016; Mishra & Narayanan, 2020; Overstreet‐Wadiche, Bromberg, Bensen, & Westbrook, 2006b; Pedroni et al., 2014; Schmidt‐Hieber et al., 2004). Second, plasticity experiments involving granule cells have revealed the manifestation of heterogeneities in the amount of synaptic plasticity induced for the same activity protocols (Beck et al., 2000; Bliss & Gardner‐Medwin, 1973; Bliss & Lomo, 1973; Davis et al., 2004; Greenstein et al., 1988; Kobayashi et al., 2013; Koranda et al., 2008; Larson & Munkacsy, 2015; McHugh et al., 2007; Pavlides et al., 1988; Shors & Dryver, 1994; Wang et al., 1997). Third, these intrinsic and plasticity heterogeneities are further amplified by the expression of adult neurogenesis. Specifically, immature adult‐born neurons manifest increased excitability, reduced synaptic connectivity, lesser dendritic arborization, and lower threshold for plasticity induction (Aimone et al., 2014; Dieni et al., 2013; Ge et al., 2007; Huckleberry & Shansky, 2021; Li et al., 2017; Lodge & Bischofberger, 2019; Schmidt‐Hieber et al., 2004). Finally, there are lines of evidence for a critical role of plasticity heterogeneity in engram formation, response decorrelation, and resource allocation in the DG. In the context of engram formation, there are postulates about the role of intrinsic excitability in governing plasticity rules and selective resource allocation (Aimone et al., 2014; Huckleberry & Shansky, 2021; Josselyn & Frankland, 2018; Josselyn & Tonegawa, 2020; Lau et al., 2020; Lodge & Bischofberger, 2019; Mishra & Narayanan, 2019; Park et al., 2016; Pignatelli et al., 2019; Yiu et al., 2014). Thus, GCs allowed us to place plasticity heterogeneity within a strong functionally relevant context of engram formation and response decorrelation. Together, GCs provided an efficient substrate for assessing the impact of well‐characterized biophysical and structural heterogeneities on the emergence of plasticity heterogeneity.

In this study, we systematically explored the cellular‐scale origins of heterogeneities in the synaptic plasticity profiles of DG GCs through an unbiased exploration of heterogeneities in their intrinsic, synaptic, and structural properties. We ensured that our analyses associated with each of these heterogeneities were constrained by characteristic physiological properties of mature and immature GCs. We assessed the impact of these forms of heterogeneities on plasticity profiles obtained with two well‐established protocols for inducing synaptic plasticity in DG GCs: the 900‐pulses protocol spanning a range of induction frequencies (Kobayashi et al., 2013; Koranda et al., 2008; Wang et al., 1997), and the theta‐burst stimulation protocol (Beck et al., 2000; Davis et al., 2004; Greenstein et al., 1988; Larson & Munkacsy, 2015; McHugh et al., 2007; Pavlides et al., 1988; Shors & Dryver, 1994). We found that each form of intrinsic, synaptic, and structural heterogeneity independently resulted in plasticity heterogeneities, with either protocol for plasticity induction. Importantly, when immature and mature neuron populations were individually analyzed, we found that heterogeneities in intrinsic excitability were insufficient to impose strong constraints on plasticity‐related measurements. However, when the entire population covering mature and immature cells were analyzed *together*, there were strong relationships between intrinsic excitability and measurements associated with synaptic plasticity.

We show that the expression of heterogeneities in all of structural, synaptic, and intrinsic neuronal properties does not necessarily have to translate to heterogeneities in synaptic plasticity profiles. Specifically, we demonstrate that very *similar* plasticity profiles could be achieved with disparate combinations of neuronal passive properties, ion‐channel properties, calcium‐handling mechanisms, synaptic strength, and neural structure of DG GCs of different ages. When observed independently, these properties manifested widespread heterogeneities with weak pairwise relationships. However, when seen together, these heterogeneities synergistically interacted with each other to achieve the functional goal of degeneracy in synaptic plasticity profiles. These analyses extend degeneracy in DG GCs to the *concomitant* emergence of plasticity profiles and of several neural intrinsic properties. Importantly, this form of degeneracy encompasses cellular‐scale intrinsic, synaptic, and structural heterogeneities spanning different age groups of GCs in a physiologically constrained manner. These analyses also showed that synaptic plasticity in the useful physiological range could be achieved in immature cells even with the weak synaptic strengths that they are known to express, owing to strong relationships with intrinsic excitability measurements.

Together, our analyses demonstrate that intrinsic, synaptic, and structural heterogeneities could either individually or through synergistic interactions among them, drive plasticity heterogeneity in DG GCs. Importantly, our analyses demonstrate that similar plasticity profiles could be achieved despite the concomitant expression of all forms of neural‐circuit heterogeneities. These observations caution against treating the manifestation of neural‐circuit heterogeneities as direct evidence for the expression of plasticity heterogeneities. Our results also highlighted the dominance of structural heterogeneities, introduced by adult neurogenesis, in introducing plasticity heterogeneity that is essential for context‐specific resource allocation in the DG. From a broader perspective, our analyses call for systematic characterization and analyses of plasticity heterogeneities across different brain regions. Such analyses should probe the mechanistic origins of plasticity heterogeneities and assess their implications for context‐specific neural coding of learned behavior and memory storage.

## 2 MATERIALS AND METHODS

Granule cells in the DG exhibit heterogeneities in neuronal properties (intrinsic heterogeneity), in synaptic connections (synaptic heterogeneity), and structural properties including dendritic arborization and surface area (structural heterogeneity). In this study, our goal is to explore the impact of these heterogeneities on synaptic plasticity profiles, employing conductance‐based models for DG GCs. Assessment of plasticity profiles involve long‐term simulations and the complexities associated with incorporating different forms of heterogeneities in *a population of conductance‐based models* (as opposed to a single model with fixed structure and fixed synaptic strengths) implied large computational costs. Thus, we employed single‐compartmental conductance‐based models to assess the impact of different forms of biophysical and structural heterogeneities on synaptic plasticity induced through two extensively employed plasticity‐induction protocols.

### 2.1 Heterogeneities in intrinsic properties of a physiologically constrained granule cell model population

**FIGURE 1:**
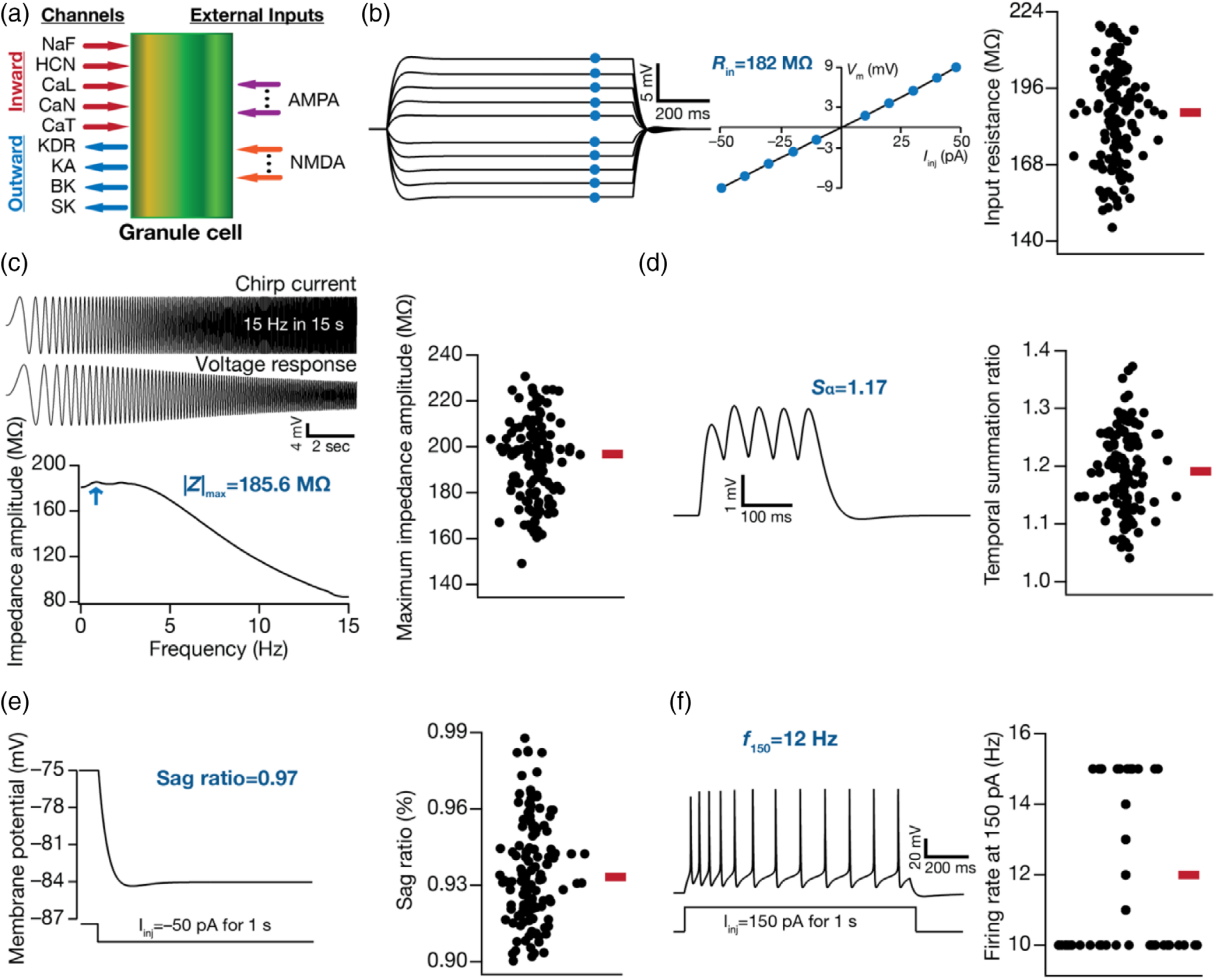
Model components of dentate gyrus granule cells and illustration of intrinsic heterogeneities across different physiological measurements. (a) Conductance‐based single compartmental model of granule cell expressing different inward and outward voltage‐dependent ion‐channel currents, receiving excitatory inputs modeled as α‐amino‐3‐hydroxy‐5‐methyl‐4‐isoxazolepropionic acid (AMPA) and *N*‐methyl‐d‐aspartate (NMDA) receptor currents. (b–f) Different intrinsic physiological measurements are employed to define the valid population of granule cells (GC) models ($N_{\mathrm{GC}}$ = 126). (b) *Left*, voltage traces in response to current pulses of amplitude −50 pA to +50 pA, in steps of 10 pA. *Right*, input resistance (*R*_in_), calculated as the slope of the *V–I* curve obtained by plotting the steady‐state voltage responses against injected current amplitudes. (c) *Top*, a chirp current stimulus of 50 pA peak‐to‐peak amplitude with linearly increasing frequency from 0 to 15 Hz in 15 s depicted along with the respective voltage response. *Bottom*, the impedance amplitude profile obtained from the chirp current and voltage response shown above. (d) *Left*, voltage response of a GC model to current input comprised five α‐EPSCs arriving at 20 Hz, to compute temporal summation ratio ($S_{\alpha }$). $S_{\alpha }$ is the ratio of the voltage amplitude in response to the fifth α‐EPSC to that of the first α‐EPSC. (e) *Left*, membrane potential in response to 50 pA hyperpolarizing current pulse to calculate sag ratio. Sag ratio is the ratio between the steady‐state voltage response and the peak voltage response. (f) *Left*, firing pattern and firing rate in response to the 150 pA depolarizing current pulse of 1 s duration. Across all panels in (b–f), the *right* panels show beeswarm plots depicting heterogeneities in the respective measurement across all 126 models. The heterogeneous population of 126 GC models employed here is from Mishra and Narayanan (2019), with additional characterization involving new intrinsic measurements added to the validation process.

Granule cells in the DG manifest pronounced heterogeneities in their intrinsic properties (Aradi & Holmes, 1999; Krueppel et al., 2011; Lubke et al., 1998; Mishra & Narayanan, 2020; Santhakumar et al., 2005). The physiologically constrained conductance‐based heterogeneous population of granule cell model was obtained from an earlier study (Mishra & Narayanan, 2019). The details of building this population of models that manifested characteristic electrophysiological properties of GCs, employing a multiparametric multiobjective stochastic search (MPMOSS) algorithm are identical to the previous study (Mishra & Narayanan, 2019). Briefly, the dimensions of single cylindrical base model were set to 63 μm diameter (*diam*) and 63 μm length (*len*) (Figure 1a). The resting membrane potential of model cell was set to −75 mV, with specific membrane resistance (*R*_m_) of 38 kΩ.cm^2^ and specific membrane capacitance (*C*_m_) of 1 μF.cm^−2^. The dimensions of the cylindrical compartment were set toward achieving a *passive* input resistance of 305 MΩ (*R*_m_/(π × diam × len) = 38 × 10^3^ × 10^−2^ × 10^−2^/(π × 63 × 10^−6^ × 63 × 10^−6^) = 305 MΩ), matching the experimental value of 309 ± 14 MΩ obtained with pharmacological blockers of HCN channels (Chen, 2004). This *passive* input resistance was consequent to the leak conductance (specified as *R*_m_) and the surface area of the compartment, and will be validated against the electrophysiological values of active input resistance (i.e., in the presence of subthreshold ion channels). These passive parameters also resulted in a charging time constant (*R*_m_ *C*_m_) of 38 ms (Schmidt‐Hieber et al., 2007).

The GC model is comprised of nine different regenerative and restorative conductances: fast sodium (NaF), hyperpolarization‐activated cyclic‐nucleotide‐gated (HCN), L‐type calcium (CaL), N‐type calcium (CaN), T‐type calcium (CaT), delayed rectifier potassium (KDR), A‐type potassium (KA), big conductance (BK), and small conductance (SK) calcium activated potassium. Hodgkin–Huxley (HH) or Goldman–Hodgkin–Katz (GHK) formulations (Goldman, 1943; Hodgkin & Huxley, 1952; Hodgkin & Katz, 1949) were employed to model these voltage‐ and/or calcium‐gated ion channels (Mishra & Narayanan, 2019). The GHK formulation was used to model calcium conductances, with intracellular and extracellular calcium concentration set at 50 nM and 2 mM, respectively. The reversal potential values for Na, K, and HCN channels were set as +55, −90, and −30 mV, respectively. Cytosolic calcium concentration and its evolution with time was dependent on calcium current and its decay, and the mechanism was adopted from the formulation (Carnevale & Hines, 2006; Destexhe et al., 1993; Narayanan & Johnston, 2010; Poirazi et al., 2003):

(1) $$\frac{d{\left[\mathrm{Ca}\right]}_{c}}{\mathrm{dt}}=-\frac{10000\cdot I_{\mathrm{Ca}}}{36\cdot \mathrm{dpt}\cdot F}+\frac{{\left[\mathrm{Ca}\right]}_{\infty }-{\left[\mathrm{Ca}\right]}_{c}}{\tau_{\mathrm{Ca}}}$$

where *F* is the Faraday's constant, the calcium decay constant in GCs was given by $\tau_{\mathrm{Ca}}$ with a default value of 160 ms, *dpt* represented the depth of the shell into which calcium influx occurred and was taken as 0.1 μm, and ${\left[\mathrm{Ca}\right]}_{\infty }$ = 50 nM was considered as the steady‐state value of ${\left[\mathrm{Ca}\right]}_{c}$.

We generated 20,000 models of GC through a stochastic search from a parametric space comprised of 40 different parameters (Table 1): 38 parameters associated with nine active conductances along with 2 passive neuronal parameters. The GC models were declared valid once they fall within the range of nine physiologically constrained measurements (Table 2): input resistance ($R_{\text{in}}$), sag ratio, firing rate at 50 pA ($f_{50}$) and 150 pA ($f_{150}$) current injection, spike frequency adaptation, action potential (AP) amplitude, AP threshold, AP half width, and fast afterhyperpolarization. The validation process resulted in 126 valid models (126/20,000, implying a 6.3% population of valid models) that manifested characteristic electrophysiological properties of GCs but exhibited pronounced heterogeneities in channel composition and other biophysical parameters (Mishra & Narayanan, 2019). This constitutes an instance of ion‐channel degeneracy (Goaillard & Marder, 2021; Mishra & Narayanan, 2019, 2021a; Rathour & Narayanan, 2019) in the emergence of cellular‐scale properties and provided 126 GC models that were endowed with signature heterogeneities in their intrinsic properties. In our analyses, this population of 126 GC models is identical to the models from Mishra and Narayanan (2019) and was employed as the substrate for assessing the impact of *intrinsic heterogeneities* on synaptic plasticity profiles.

**TABLE 1:** The multiple parameters and their ranges for the stochastic search employed for finding the 126 valid granule cells (Mishra & Narayanan, 2019).

	Parameters	Symbol	Default	Testing range
*h* channel properties				
1	Maximal conductance (μS/cm^2^)	*h‐g*	5	2–2
2	Activation time constant of *I*_h_ (ms)	*h‐τ*_A_	39	30–50
3	*V*_1/2_ activation of *I*_h_ (mV)	*h‐V*_A_	–81	−70 to −90
*A*‐type K^+^ channel properties				
4	Maximal conductance (mS/cm^2^)	*KA‐g*	87	70–110
5	Activation time constant of KA (ms)	*KA‐τ*_A_	0.454	0.42–0.7
6	Inactivation time constant of KA (ms)	*KA‐τ*_I_	6.54	3–10
7	*V*_1/2_ activation of KA (mV)	*KA‐V*_A_	−55	−50 to −62
8	*V*_1/2_ inactivation of KA (mV)	*KA‐V*_I_	−73.1	−69 to −82
Delayed rectifier K^+^ channel properties				
9	Maximal conductance (μS/cm^2^)	*KDR‐g*	500	320–1100
10	Activation time constant of KDR (ms)	*KDR‐τ*_A_	6.4	5–10
11	*V*_1/2_ activation of KDR (mV)	*KDR‐V*_A_	−44	−38 to −50
Fast Na^+^ channel properties				
12	Maximal conductance (mS/cm^2^)	*Na‐g*	18	16–50
13	Activation time constant of Na (μs)	*Na‐τ*_A_	50	42–56
14	Inactivation time constant of Na (ms)	*Na‐τ*_I_	3	2–6
15	*V*_1/2_ activation of Na (mV)	*Na‐V*_A_	−31	−30 to −40
16	*V*_1/2_ inactivation of Na (mV)	*Na‐V*_I_	−49	−43 to −55
Small conductance Ca^2+^−dependent potassium (*SK*) channel properties				
17	Maximal conductance (mS/cm^2^)	*SK‐g*	5	1–12
18	*Ca*_1/2_ activation of SK (μM)	*SK‐C*_A_	4	1–8
19	Activation time constant of SK (ms)	*SK‐τ*_A_	214	195–250
20	Decay constant of calcium	*Ca‐τ*_decay_	160	95–206
Large conductance Ca^2+^−activated potassium (*BK*) channel properties				
21	Maximal conductance (mS/cm^2^)	*BK‐g*	110	14–190
22	*Ca*_1/2_ activation of BK (μM)	*BK‐C*_A_	4	2–7
23	Activation time constant of BK (Ca^2+^ dependent) (ms)	*BK‐Cτ*_A_	10	5–15
24	Activation time constant of BK (voltage dependent) (μs)	*BK‐τ*_A_	5	3–11
25	*V*_1/2_ activation of BK (mV)	*BK‐V*_A_	−28	−18 to −36
*L*‐type Ca^2+^ channel properties				
26	Maximal conductance (μS/cm^2^)	*CaL‐g*	700	105–800
27	Activation time constant of *L*‐type (μs)	*CaL‐τ*_A_	3	1–12
28	*V*_1/2_ activation of *L*‐type (mV)	*CaL‐V*_A_	−1.3	−5 to 7
*N*‐type Ca^2+^ channel properties				
29	Maximal conductance (μS/cm^2^)	*CaN‐g*	0.5	0.1–5
30	Activation time constant of *N*‐type (ms)	*CaN‐τ*_A_	0.6	0.1–1
31	Inactivation time constant of *N*‐type (ms)	*CaN‐τ*_I_	1297	1050–1450
32	*V*_1/2_ activation of *N*‐type (mV)	*CaN‐V*_A_	−21	−30 to −10
33	*V*_1/2_ inactivation of *N*‐type (mV)	*CaN‐V*_I_	−40	−50 to −30
*T*‐type Ca^2+^ channel properties				
34	Maximal conductance (μS/cm^2^)	*CaT‐g*	0.7	0.5–10
35	Activation time constant of *T*‐type (ms)	*CaT‐τ*_A_	4	2–10
36	Inactivation time constant of *T*‐type (ms)	*CaT‐τ*_I_	7665	6800–8400
37	*V*_1/2_ activation of *T*‐type (mV)	*CaT‐V*_A_	−36	−28 to −42
38	*V*_1/2_ inactivation of *T*‐type (mV)	*CaT‐V*_I_	−67	−75 to −58
Passive properties				
39	Specific membrane resistivity (kΩ.cm^2^)	*R*_m_	38	30–42
40	Specific membrane capacitance (μF/cm^2^)	*C*_m_	1	0.8–1.2

**TABLE 2:** Electrophysiological bounds for the multiple objectives, defining characteristic granule cell measurements, of the stochastic search procedure that spanned 20,000 independent samples on the parameters in Table 1 (Mishra & Narayanan, 2019).

	Measurement, unit	Symbol	Lower	Upper
1	Action potential amplitude, mV	*V*_AP_	95	115
2	Action potential threshold, mV	*V*_th_	−55	−40
3	Action potential half‐width, ms	*T*_APHW_	0.53	1.6
4	Fast after hyperpolarization, mV	*V*_fAHP_	−25	−3.4
5	Sag ratio	Sag ratio	0.9	1
6	Spike frequency adaptation	SFA	0.1	0.8
7	Input resistance, MΩ	*R*_in_	107	228
8	Firing frequency at 50 pA, Hz	*f*_50_	0	0
9	Firing frequency at 150 pA, Hz	*f*_150_	10	15
10	Temporal summation ratio	$S_{\alpha }$	0.92	2.12
11	Maximum impedance amplitude, MΩ	*|Z|*_max_	63.4	430.2

*Note*: The first nine measurements were employed to validate the 126 (of the 20,000 samples) intrinsically heterogeneous model neurons (Mishra & Narayanan, 2019), whereas the last two measurements were validated for the 126 models (Figure 1) with electrophysiological bounds derived from Mishra and Narayanan (2020). These 126 models showed characteristic electrophysiological properties and neuron‐to‐neuron heterogeneity that were comparable with electrophysiological recordings (Mishra & Narayanan, 2019, 2020, 2021a). These 126 valid models were sufficient to demonstrate that disparate combinations of ion channels could yield very similar characteristic properties (Mishra & Narayanan, 2019, 2021b). Importantly, the parametric values of these 126 models spanned the entire valid range of each parameter suggesting the absence of any parametric clustering (Mishra & Narayanan, 2019), together demonstrating the expression of ion‐channel degeneracy (Mishra & Narayanan, 2021a).

### 2.2 Properties and associated heterogeneities in synapses impinging on granule cell models

We modeled a canonical synapse impinging on the postsynaptic GC neuron as two co‐localized excitatory synaptic receptors: α‐amino‐3‐hydroxy‐5‐methyl‐4‐isoxazolepropionic acid (AMPA) receptor (AMPAR) and *N*‐methyl‐d‐aspartate (NMDA) receptor (NMDAR) with an NMDA: AMPA ratio value of 1.5. The current through AMPAR and NMDAR as a function of voltage and time are modeled using the GHK formulation (Goldman, 1943; Hodgkin & Katz, 1949) as a sum of current generated by sodium and potassium ions (Anirudhan & Narayanan, 2015; Honnuraiah & Narayanan, 2013; Narayanan & Johnston, 2010):

(2) $$I_{\text{AMPAR}}\left(v,t\right)=I_{\text{AMPAR}}^{\mathrm{Na}}\left(v,t\right)+I_{\text{AMPAR}}^{K}\left(v,t\right),$$

where

(3) $$I_{\text{AMPAR}}^{\mathrm{Na}}\left(v,t\right)={\bar{P}}_{\text{AMPAR}}\;w\;P_{\mathrm{Na}}\;s\left(t\right)\;\frac{vF^{2}}{\mathrm{RT}}\;\left(\frac{{\left[\mathrm{Na}\right]}_{i}-{\left[\mathrm{Na}\right]}_{o}\exp\left(-\frac{\mathrm{vF}}{\mathrm{RT}}\right)}{1-\exp\left(-\frac{\mathrm{vF}}{\mathrm{RT}}\right)}\right),$$

(4) $$I_{\text{AMPAR}}^{K}\left(v,t\right)={\bar{P}}_{\text{AMPAR}}\;w\;P_{K}\;s\left(t\right)\;\frac{vF^{2}}{\mathrm{RT}}\;\left(\frac{{\left[K\right]}_{i}-{\left[K\right]}_{o}\exp\left(-\frac{\mathrm{vF}}{\mathrm{RT}}\right)}{1-\exp\left(-\frac{\mathrm{vF}}{\mathrm{RT}}\right)}\right).$$

Here, ${\bar{P}}_{\text{AMPAR}}$ represents the maximum permeability of the receptor, also used as a synaptic parameter to incorporate synaptic heterogeneity. w represents the synaptic weight parameter that would be updated and monitored as a function of time to quantify positive and negative weight changes based on the plasticity protocol (see below). The default value of initial weight, $w_{\text{init}}$ was set to 0.25. The sodium ($P_{\mathrm{Na}}$) and potassium ($P_{K}$) permeability values were set to be equal ($P_{\mathrm{Na}}$: $P_{K}$ = 1:1) based on experimental observations. The default values for intracellular and extracellular concentration (mM) of specific ions were ${\left[\mathrm{Na}\right]}_{i}$ = 18, ${\left[\mathrm{Na}\right]}_{o}$ = 140, ${\left[K\right]}_{i}$ = 140, ${\left[K\right]}_{o}$ = 5, which led to equilibrium potential of +55 mV and −90 mV for Na and K, respectively. $s\left(t\right)$ guides the kinetics of AMPA current as represented using the two‐exponential formulation:

(5) $$s\left(t\right)=a\left(\exp\left(-\frac{t}{\tau_{d}}\right)-\exp\left(-\frac{t}{\tau_{r}}\right)\right)$$

where a represents normalization constant so that 0 < *s*(*t*) < 1. $\tau_{r}$ and $\tau_{d}$ denote the rise and decay time constants associated with AMPA receptor with values of 2 and 10 ms, respectively. *Synaptic heterogeneities* were introduced into the population of models by altering the permeability value of ${\bar{P}}_{\text{AMPAR}}$.

The current through NMDA receptor depended on sodium, potassium, and calcium ions and was modeled as follows using the GHK formulation:

(6) $$I_{\text{NMDAR}}\left(v,t\right)=I_{\text{NMDAR}}^{\mathrm{Na}}\left(v,t\right)+I_{\text{NMDAR}}^{K}\left(v,t\right)+I_{\text{NMDAR}}^{\mathrm{Ca}}\left(v,t\right)$$

where

(7) $$I_{\text{NMDAR}}^{\mathrm{Na}}\left(v,t\right)={\bar{P}}_{\text{NMDAR}}\;P_{\mathrm{Na}}\;s\left(t\right)\;\mathrm{MgB}\left(v\right)\;\frac{vF^{2}}{\mathrm{RT}}\;\left(\frac{{\left[\mathrm{Na}\right]}_{i}-{\left[\mathrm{Na}\right]}_{o}\exp\left(-\frac{\mathrm{vF}}{\mathrm{RT}}\right)}{1-\exp\left(-\frac{\mathrm{vF}}{\mathrm{RT}}\right)}\right)$$

(8) $$I_{\text{NMDAR}}^{K}\left(v,t\right)={\bar{P}}_{\text{NMDAR}}\;P_{K}\;s\left(t\right)\;\mathrm{MgB}\left(v\right)\;\frac{vF^{2}}{\mathrm{RT}}\;\left(\frac{{\left[K\right]}_{i}-{\left[K\right]}_{o}\exp\left(-\frac{\mathrm{vF}}{\mathrm{RT}}\right)}{1-\exp\left(-\frac{\mathrm{vF}}{\mathrm{RT}}\right)}\right),$$

(9) $$I_{\text{NMDAR}}^{\mathrm{Ca}}\left(v,t\right)={\bar{P}}_{\text{NMDAR}}\;P_{\mathrm{Ca}}\;s\left(t\right)\;\mathrm{MgB}\left(v\right)\;\frac{4vF^{2}}{\mathrm{RT}}\;\left(\frac{{\left[\mathrm{Ca}\right]}_{i}-{\left[\mathrm{Ca}\right]}_{o}\exp\left(-\frac{2\mathrm{vF}}{\mathrm{RT}}\right)}{1-\exp\left(-\frac{2\mathrm{vF}}{\mathrm{RT}}\right)}\right).$$

${\bar{P}}_{\text{NMDAR}}$ denotes the maximum permeability of the NMDA receptor and was defined as the product of ${\bar{P}}_{\text{AMPAR}}$, $w_{\text{init}}$, and the value of NMDA:AMPA ratio. The permeability ratios of three ions for NMDAR are set as $P_{\mathrm{Ca}}:P_{\mathrm{Na}}:P_{K}$ = 10.6:1:1 (Canavier, 1999; Mayer & Westbrook, 1987). The $s\left(t\right)$ function was same as for AMPAR with $\tau_{r}$ = 5 ms and $\tau_{d}$ = 50 ms. The concentration values in mM are ${\left[\mathrm{Na}\right]}_{i}$ = 18, ${\left[\mathrm{Na}\right]}_{o}$ = 140, ${\left[K\right]}_{i}$ = 140, ${\left[K\right]}_{o}$ = 5 ${\left[\mathrm{Ca}\right]}_{i}$ = 100 × 10^−6^, and ${\left[\mathrm{Ca}\right]}_{o}$ = 2. $\mathrm{Mg}B\left(v\right)$ refers to the sigmoidal (${\left(1+{\left[\mathrm{Mg}\right]}_{o}\exp\left(–0.062\;v\right)/3.57\right)}^{-1}$) dependence of NMDAR currents on extracellular magnesium concentration (${\left[\mathrm{Mg}\right]}_{o}$) and voltage (Jahr & Stevens, 1990). The current through NMDAR did not undergo plasticity.

### 2.3 Heterogeneities in structural properties of the granule cell population

*Structural heterogeneities*, mediated by the expression of adult neurogenesis in the DG, were incorporated into the GC model population by subjecting the mature set of 126 valid models to structural plasticity. Specifically, the reduction in dendritic arborization and in the overall number of channels expressed in immature neurons (Aimone et al., 2014) was approximated by a reduction in the diameter of the model neuron, using *R*_in_ as the measurement to match with experimental counterparts (Mishra & Narayanan, 2019). Electrophysiologically, *R*_in_ of mature and immature cells have been measured to be in the ~100–300 MΩ and ~3–6 GΩ ranges, respectively (Heigele et al., 2016; Mishra & Narayanan, 2020, 2021a; Overstreet‐Wadiche, Bromberg, et al., 2006; Pedroni et al., 2014; Schmidt‐Hieber et al., 2004). Reducing the diameter of the models in neural population increased neuronal excitability, reflecting as increased *R*_in_ and increased firing rate. To assess the impact of structural heterogeneities on synaptic plasticity profiles, we varied the diameter of the 126 neurons in the model population from 1 to 63 μm. A diameter range of 2–9 μm was chosen because this yielded *R*_in_ values that matched the experimental 3–6 GΩ range for immature neurons and was considered representative of the immature neuronal models (Mishra & Narayanan, 2019).

### 2.4 Intrinsic measurements

The 126 GC models were selected based on the nine physiological measurements employed to characterize the valid GC population (Table 2; Mishra & Narayanan, 2019). In addition to these, we introduced two more sub‐threshold measurements (impedance amplitude and temporal summation ratio) to test the robustness of these intrinsically heterogeneous models (Figure 1c,d) and to compare their role in regulating plasticity profiles. Specifically, we employed input resistance (*R*_in_), firing frequency to pulse current injections, sag ratio, impedance amplitude, and temporal summation as intrinsic measurements towards relating them to plasticity profiles. *R*_in_ was measured as the slope of a linear fit to the *I‐V* plot. The *I‐V* plot was obtained by plotting the steady state value of voltage response as a function of 11 different current pulses where the amplitude varied from −50 to +50 pA in steps of 10 pA (Figure 1b). As GC models with lower diameters manifested high excitability, *R*_in_ was computed in response to hyperpolarizing current pulses ranging from −50 to 0 pA in steps of 10 pA, to avoid spike generation. To characterize the impedance amplitude profiles of these models, we injected chirp stimulus, a frequency‐dependent current input with linearly increasing frequency from 0 to 15 Hz in 15 s of constant amplitude (Mishra & Narayanan, 2020). The impedance profile $Z\left(f\right)$ was computed as the ratio of the Fourier transform of voltage response to the Fourier transform of chirp current as a function of frequency (Figure 1c). The impedance amplitude profile was calculated as follows:

(10) Zf=ReZf2+ImZf2,

where ReZf and ImZf refer to the real and imaginary parts of the impedance $Z\left(f\right)$, respectively, as functions of the frequency *f*. The maximum value of impedance across all frequencies was measured as the maximum impedance amplitude (|*Z*|_max_).

Temporal summation ratio ($S_{\alpha }$) was computed by injecting current pattern following the $I_{\alpha }\left(t\right)=I_{\max}\;t\exp\left(-\alpha t\right)$ formulation, where α = 0.1 ms^−1^. Five such current pulses were injected into the neuron with 50 ms interval between them, together resulting in a response consisting of five α excitatory postsynaptic potentials (α–EPSPs). The ratio of amplitude of last to first EPSP ($E_{\text{last}}/E_{\text{first}}$) was defined as the temporal summation ratio, $S_{\alpha }$ (Figure 1d). Sag ratio was computed as the ratio between the steady‐state voltage deflection to the peak voltage deflection from *V*_RMP_ in response to hyperpolarizing current pulse of 50 pA injected for a period of 1 s (Figure 1e). The firing property of GC models was characterized by computing the firing rate in response to a current pulse of 100 pA ($f_{100}$) or 150 pA ($f_{150}$) for 1 s (Figure 1f).

### 2.5 Synaptic plasticity protocols and weight evolution

The synaptic weight parameter w governing current through AMPAR depended on the intracellular calcium concentration as follows, based on the calcium control hypothesis (Shouval et al., 2002):

(11) $$\frac{\mathrm{dw}}{\mathrm{dt}}=\eta \left({\left[\mathrm{Ca}\right]}_{i}\right)\left(\Omega \left({\left[\mathrm{Ca}\right]}_{i}\right)–w\right),$$

where $\eta \left({\left[\mathrm{Ca}\right]}_{i}\right)$ represents learning rate dependent on calcium concentration, which is inversely related to learning time constant $\tau \left({\left[\mathrm{Ca}\right]}_{i}\right)$ as follows:

(12) $$\eta \left({\left[\mathrm{Ca}\right]}_{i}\right)=\frac{1}{\tau \left({\left[\mathrm{Ca}\right]}_{i}\right)},$$

(13) $$\tau \left({\left[\mathrm{Ca}\right]}_{i}\right)=P_{1}+\frac{P_{2}}{P_{3}+{\left[\mathrm{Ca}\right]}_{i}^{P_{4}}},$$

where *P*_1_ = 1 s, *P*_2_ = 0.1 s, *P*_3_ = *P*_2_ × 10^−4^, and *P*_4_ = 3. These values when substituted in Equation (12) sets the learning time constant to ~3 h when ${\left[\mathrm{Ca}\right]}_{i}$ is ~0. $\Omega \left({\left[\mathrm{Ca}\right]}_{i}\right)$, the function that governed the calcium‐dependent weight update mechanism, was defined as (Shouval et al., 2002):

(14) $$\Omega \left({\left[\mathrm{Ca}\right]}_{i}\right)=0.25+\frac{1}{1+\exp\left(-\beta_{2}\left({\left[\mathrm{Ca}\right]}_{i}-\alpha_{2}\right)\right)}-0.25\left(\frac{1}{1+\exp\left(-\beta_{1}\left({\left[\mathrm{Ca}\right]}_{i}-\alpha_{1}\right)\right)}\right)$$

where *α*_1_ = 0.35, *α*_2_ = 0.55, *β*_1_ = *β*_2_ = 80. For all the weight update equations, ${\left[\mathrm{Ca}\right]}_{i}$ were set as the deflection from the resting value of ${\left[\mathrm{Ca}\right]}_{i}$.

**FIGURE 2:**
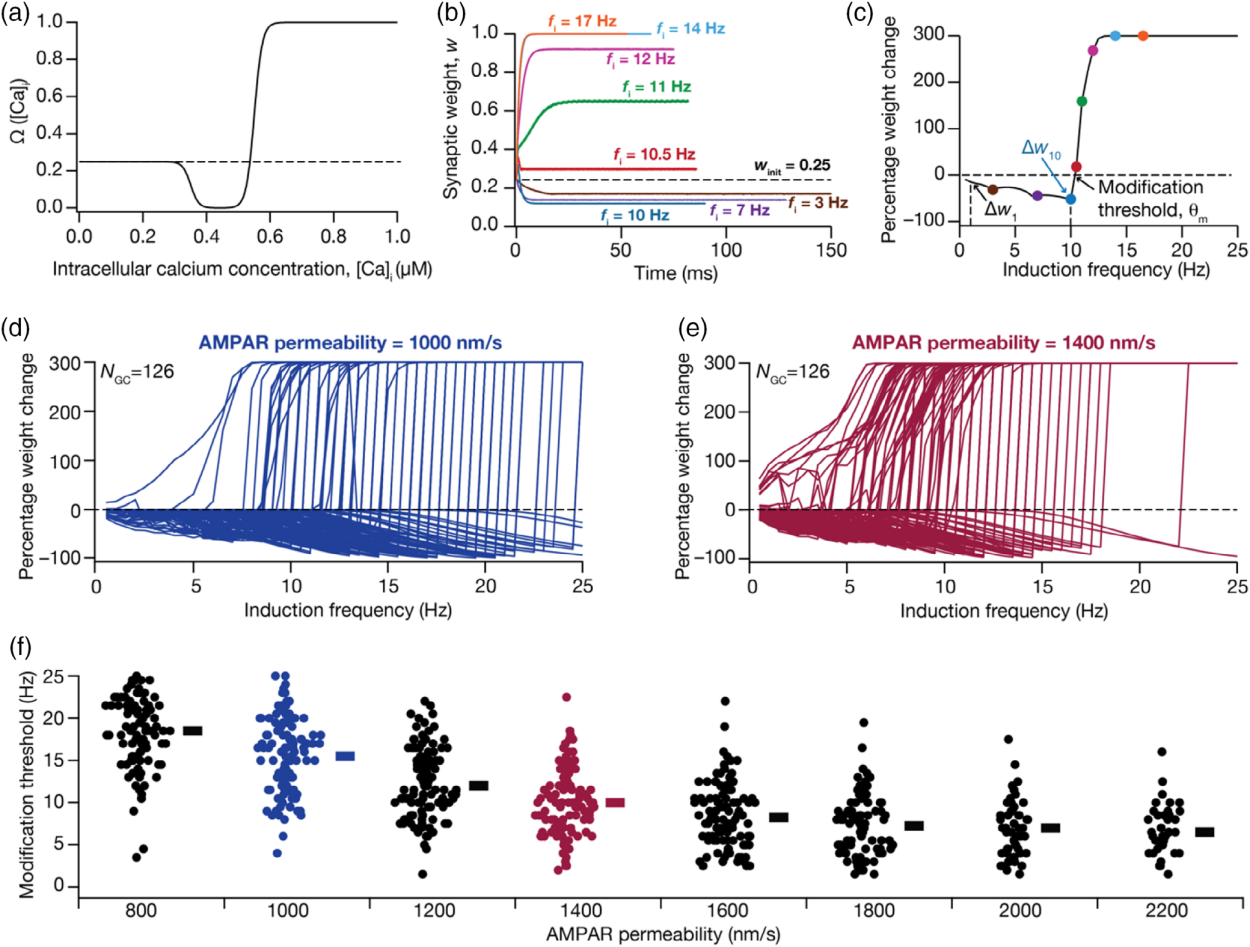
Intrinsic heterogeneities in the granule cell population translates to heterogeneities in their BCM‐like synaptic plasticity profiles, when synaptic properties were fixed across models. (a) Plot of the Ω‐function based on the calcium control hypothesis that regulates level of plasticity as a function of intracellular Ca^2+^ concentration (Equation 11). (b) Evolution of synaptic weight as a function of time, obtained by employing 900‐pulses protocol of different induction frequencies in a granule cell (GC) model. Note that all plots initialize at $w_{\text{init}}=0.25$ and evolve to reach their respective steady‐state value. The duration of each plot spans 900 pulses at the specified induction frequency $f_{i}$. (c) BCM‐like synaptic plasticity profile obtained by plotting the percentage change in synaptic weight parameter after stimulation with 900‐pulses of different induction frequencies ranging from 0.5 to 25 Hz. The color‐coded points correspond to the different induction frequencies shown in panel b. Arrows point to $\theta_{m}$, $\Delta w_{1}\;$ and $\Delta w_{10}$. $\Delta w_{1}$ and $\Delta w_{10}$ represent the change in synaptic weight value for induction frequencies of 1 and 10 Hz, respectively; $\theta_{m}$, the modification threshold, is the induction frequency at which the plasticity profile switches from inducing LTD to inducing LTP. (d–e) Same as (c), for all the 126 GC models for two different values of α‐amino‐3‐hydroxy‐5‐methyl‐4‐isoxazolepropionic acid receptor (AMPAR) permeability: 1000 nm/s (d) and 1400 nm/s (e). (f) Beeswarm plots of modification threshold for all GC models, for different values of AMPAR permeability. Note that with specific values of AMPAR permeability, there were models that did not manifest a $\theta_{m}$ in the tested range of frequencies, thus resulting in lesser number of models for each AMPAR permeability values (*N* = 100, 121, 121, 120, 112, 94, 63, 39 left to right).

Using this framework, we analyzed the direction and strength of plasticity in *w* using two well‐established synaptic plasticity protocols in DG neurons: the 900‐pulses protocol with varying induction frequencies (Kobayashi et al., 2013; Koranda et al., 2008; Wang et al., 1997) and the theta burst stimulation (TBS) protocol (Beck et al., 2000; Davis et al., 2004; Greenstein et al., 1988; Larson & Munkacsy, 2015; McHugh et al., 2007; Pavlides et al., 1988; Shors & Dryver, 1994). The 900‐pulses protocol involved synaptic stimulation made up of 900 pulses at various induction frequencies (*f*_i_ spanning 0.5–25 Hz), an experimentally and computationally well‐established Bienenstock–Cooper–Munro (BCM)‐like (Bienenstock et al., 1982) plasticity protocol across different neurons including DG GCs (Anirudhan & Narayanan, 2015; Ashhad & Narayanan, 2013; Cooper & Bear, 2012; Dudek & Bear, 1992; Honnuraiah & Narayanan, 2013; Johnston et al., 2003; Kobayashi et al., 2013; Koranda et al., 2008; Narayanan & Johnston, 2010; Shouval et al., 2002; Wang et al., 1997). The evolution of synaptic weight (Equation 11) was monitored and the final weight at the end of the induction protocol was plotted as a function of the induction frequency (Figure 2b). The percentage difference between this final weight and the initial weight (0.25) was plotted against the induction frequency of the stimulus pulses to obtain the synaptic plasticity profile (Figure 2c) as a function of induction frequency (Anirudhan & Narayanan, 2015; Honnuraiah & Narayanan, 2013; Narayanan & Johnston, 2010; Shouval et al., 2002). The induction frequency at which this plasticity profile transitioned from depression to potentiation was defined as the modification threshold (Figure 2c), $\theta_{m}$ (Ashhad & Narayanan, 2013; Cooper & Bear, 2012; Dudek & Bear, 1992; Honnuraiah & Narayanan, 2013; Johnston et al., 2003; Narayanan & Johnston, 2010; Shouval et al., 2002).

We also employed percentage changes in w with $f_{i}$ = 1 Hz ($\Delta w_{1}$) and $f_{i}$ = 10 Hz ($\Delta w_{10}$) for quantifying synaptic plasticity profiles (Figure 2c). The computational complexity of this process was enormous, especially in the face of three different forms of heterogeneities, and given that the construction of each profile required stimulating the synapses with 900 pulses for each of the 50 induction frequencies ($f_{i}$ spanning 0.5–25 Hz; 0.5 Hz increment) for each of the 126 models, across several synaptic permeability and diameter values. This complexity was essential in assessing the mechanistic origins of plasticity heterogeneity through a systematic and unbiased methodology, incorporating different forms of neural‐circuit heterogeneities in a physiologically constrained manner, rather than employing a single hand‐tuned model with predetermined assumptions about the role of individual components.

For TBS, the synapse was stimulated with a burst of five action potentials at 100 Hz, and this burst was repeated 150 times at 200 ms interval (theta frequency) each (Figure 5a). This was done to achieve steady‐state values for ${\left[\mathrm{Ca}\right]}_{i}$ and *w* (Ashhad & Narayanan, 2013). The percentage change in w at the end of this protocol in comparison to its initial value ($w_{\text{init}}=0.25$) was employed to quantify plasticity induced with TBS. For both plasticity induction protocols, we employed a spike train generator as an input source to mimic presynaptic activity.

These synaptic plasticity protocols and the rules for updating synapses were chosen from the perspective of their relevance to synapses in the DG GCs. Specifically, the two protocols employed here are well‐established routes to induce synaptic plasticity in DG GCs (Beck et al., 2000; Davis et al., 2004; Greenstein et al., 1988; Kobayashi et al., 2013; Koranda et al., 2008; Larson & Munkacsy, 2015; McHugh et al., 2007; Pavlides et al., 1988; Shors & Dryver, 1994; Wang et al., 1997). The calcium‐dependent plasticity rule employed here is a BCM‐like plasticity rule that has been effectively used across different cell types to assess physiological plasticity (Anirudhan & Narayanan, 2015; Ashhad & Narayanan, 2013; Bienenstock et al., 1982; Castellani et al., 2001; Castellani et al., 2005; Cooper & Bear, 2012; Dudek & Bear, 1992; Honnuraiah & Narayanan, 2013; Magee & Grienberger, 2020; Narayanan & Johnston, 2010; Philpot et al., 2001; Shah et al., 2006; Shouval et al., 2002; Yeung et al., 2004; Yu et al., 2008). The rationale behind our choice of the calcium‐control hypothesis is the match between the plasticity profile obtained with the 900‐pulses protocol in DG GCs (Kobayashi et al., 2013; Koranda et al., 2008; Wang et al., 1997) and the calcium‐dependent plasticity profile explained by the BCM rule (Bienenstock et al., 1982; Cooper & Bear, 2012; Shouval et al., 2002).

### 2.6 Computer simulations and analysis

We employed NEURON as the simulation environment (Carnevale & Hines, 2006) for executing all the simulation at $V_{\mathrm{RMP}}$ (−75 mV) with fixed temperature set at 34°C. We used the integration time step of 25 μs except for simulations involving 900‐pulses protocol, where a variable time step method was employed to efficiently solve the associated differential equations with lower computational time. All data analyses were performed using custom‐built software under the Igor‐Pro programming environment (Wavemetrics Inc., USA). To avoid ambiguities arising from reporting merely the summary statistics (Marder & Taylor, 2011; Rathour & Narayanan, 2019), we have reported all the data points with their respective ranges to represent the heterogeneities associated with our analysis and results. As we have employed Pearson correlation coefficient for pairwise scatter plots, qualitative descriptions on the strength of correlation coefficient values (weak vs. strong) were adopted from the definitions provided in the study by Evans (1996).

## 3 RESULTS

We employed a physiologically realistic conductance‐based population of GC models ($N_{\mathrm{GC}}$=126), endowed with *intrinsic heterogeneities* and expressing ion‐channel degeneracy at the cellular‐scale (Mishra & Narayanan, 2019), to assess the impact of neural heterogeneities on synaptic plasticity profiles. In this population, we introduced *synaptic heterogeneities* by altering afferent synaptic strength, and *structural heterogeneities* by changing the surface area of the model population. We employed two well‐established synaptic plasticity protocols, namely the BCM‐like 900‐pulses protocol with different induction frequencies and the TBS protocol, to examine the impact of these three forms of neural heterogeneities in the regulation of synaptic plasticity rules in DG GCs. We present results obtained through systematic incorporation of these different forms of heterogeneities, both independently and synergistically, into a physiologically validated GC model population.

### 3.1 GC models showed robustness for nonvalidated measurements and manifested heterogeneities in intrinsic measurements

The 126 GC models employed in this study were derived from an unbiased stochastic search spanning 40 parameters (Table 1), sampling 20,000 randomized models (Mishra & Narayanan, 2019). Of the 20,000 models, these 126 models were previously validated based on nine different characteristic electrophysiological signatures (Table 2) of DG GCs (Mishra & Narayanan, 2019). Prominent among these measurements are input resistance (*R*_in_, range 140–225 MΩ; Figure 1b), sag ratio (range 0.9–1; Figure 1e) and firing rate at 150 pA (range 10–15 Hz; Figure 1f), which manifested heterogeneities. In addition to these, here we characterized two more experimentally obtained sub‐threshold measurements of excitability to assess their relationship to the induction of synaptic plasticity: impedance amplitude and temporal summation ratio (Figure 1c,d). Whereas temporal summation of postsynaptic potentials constitutes an important measurement that governs calcium influx and thereby synaptic plasticity (Narayanan & Johnston, 2010; Nolan et al., 2004), impedance is a measure of excitability for time‐varying signals (Narayanan & Johnston, 2008). Although the 126 GC models were initially not validated against these two measurements, here we found that these measurements in the models were within the range of their electrophysiological counterparts (Mishra & Narayanan, 2020). Specifically, maximum impedance amplitude (|*Z*|_max_) in the model population ranged from 149.1 to 230.8 MΩ (mean ± SEM: 194.2 ± 1.6; *N*_GC_ = 126; Figure 1c), which was within the measured electrophysiological range (Mishra & Narayanan, 2020) of 63.4–430.2 MΩ (mean ± SEM: 176.9 ± 5.3; *N* = 172). The temporal summation ratio in the model population ranged from 1.04 to 1.37 (mean ± SEM: 1.19 ± 0.006; *N*_GC_ = 126; Figure 1d), which was within the measured electrophysiological range (Mishra & Narayanan, 2020) of 0.92–2.12 (mean ± SEM: 1.33 ± 0.015; *N* = 133). Apart from providing validation against two additional intrinsic measurements, our analyses showed that these intrinsic properties manifested heterogeneities in the model population (Figure 1c,d) and provided further evidence for the robustness of our models in matching characteristic signatures of GCs. In addition, the parameters (spanning active and passive neural properties; Table 1) underlying these 126 models manifested considerable heterogeneities, thus providing a layer of biophysical heterogeneities in the GC population.

### 3.2 Intrinsic heterogeneity resulted in heterogeneities in BCM‐like plasticity profiles when models received identical synaptic inputs

To understand the impact of intrinsic heterogeneities on emergence of plasticity profiles, we first employed the well‐established BCM‐like 900 pulses protocol and constructed the synaptic plasticity profile, spanning different induction frequencies ($f_{i}$), for each GC model. The stimuli comprised 900 synaptic stimulations impinging on a synapse on each model neuron, at different induction frequencies ranging from 0.5 to 25 Hz. The synaptic stimulation was allowed to activate a synapse endowed with co‐localized AMPAR and NMDAR, with identical values for receptors' densities and properties across all GC models. Activation of these receptors resulted in influx of calcium into the cytosol, through NMDARs and voltage‐gated calcium channels (VGCC) expressed in the models, with the strength and the dynamics of calcium evolution depending upon the induction frequency and the specific model under consideration. Although the synaptic properties and stimulation protocols were identical across models, the cytosolic calcium influx would be model‐dependent because of the differential parametric configurations across models. The influx of calcium, in turn, affected the weight parameter (*w*) associated with the AMPARs (Narayanan & Johnston, 2010; Shouval et al., 2002), following the calcium control hypothesis (Figure 2a; Equation 11). We monitored the temporal evolution of the weight parameter and recorded the final value at the end of protocol (after 900 pulses) for each induction frequency (Figure 2b). To obtain the synaptic plasticity profile, the percentage weight change in *w* was computed from its final value for each $f_{i}$ with respect to the initial value ($w_{\text{init}}$ = 0.25) and was plotted as a function of $f_{i}$ (Figure 2c). Consistent with experimental results from DG GCs (Kobayashi et al., 2013; Koranda et al., 2008; Wang et al., 1997) and with the Ω‐function that governs synaptic plasticity (Figure 2a), we found that lower and higher values of $f_{i}$ yielded depression and potentiation, of AMPAR weight, respectively (Figure 2c). The induction frequency at which the synaptic plasticity profile transitioned from depression to potentiation was termed the modification threshold $\theta_{m}$ (Anirudhan & Narayanan, 2015; Bienenstock et al., 1982; Honnuraiah & Narayanan, 2013; Narayanan & Johnston, 2010; Shouval et al., 2002). The $w_{\text{init}}$ value along with the definition of the Ω‐function implies that the percentage plasticity varies from −100% to +300%, with negative sign representing depression and the positive sign representing potentiation (Narayanan & Johnston, 2010; Shouval et al., 2002).

To understand how and to what extent intrinsic heterogeneities impact the evolution of synaptic profiles and modification threshold, we obtained plasticity profiles for all the 126 GC models with *identical* structural and synaptic properties. We found that heterogeneities in intrinsic properties of these models resulted in heterogeneities in the BCM‐like plasticity profiles (Figure 2d,e) as well as in the associated modification thresholds (Figure 2f). We repeated these analyses for different values of baseline synaptic strength (defined as receptor permeability, ${\bar{P}}_{\text{AMPAR}}$, within the GHK formulation for AMPARs) to explore the association of the fixed synaptic parameter to intrinsic heterogeneities in altering the plasticity profiles (Figure 2d–f). We observed a graded reduction in the modification threshold (Figure 2f), implying a leftward shift in the BCM‐like plasticity profile, with increase in the baseline synaptic strength. This is to be expected because with increased synaptic strength, the postsynaptic depolarization and consequently the cytosolic calcium influx are higher, thus allowing the plasticity profile to transition to synaptic potentiation at lower induction frequencies (Narayanan & Johnston, 2010; Shouval et al., 2002). As a consequence of intrinsic heterogeneities across models and such leftward shifts in plasticity profile, there was also an increase in the number of models that manifested no synaptic depression (within the tested range of $f_{i}$) with increases in baseline synaptic strength. Together, these results demonstrated that the expression of intrinsic heterogeneities led to heterogeneities in synaptic plasticity profiles, when structural and synaptic properties across models were identical.

### 3.3 Weak pairwise correlations between intrinsic and plasticity‐profile measurements

**FIGURE 3:**
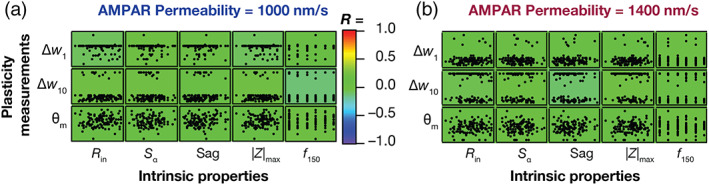
Weak pairwise correlations between measurements of synaptic plasticity and intrinsic properties in the heterogeneous granule cells (GC) model population. (a,b) Pairwise scatter plot matrices between three plasticity measurements: Percentage weight change at 1 Hz ($\Delta w_{1}$), 10 Hz ($\Delta w_{10}$) and the modification threshold ($\theta_{m}$) along the vertical axes, and five intrinsic measurements: *R*_in_, $S_{\alpha }$, *f*_150_, |*Z*|_max_, and sag on the horizontal axes. Synaptic plasticity measurements were obtained for baseline AMPAR permeability values of 1000 nm/s (a) and 1400 nm/s (b). The scatter plot matrices are overlaid on the respective color‐coded values of correlation coefficients (*R*).

How are the different intrinsic measurements defining the 126 GC models related to the measurements employed to quantify the synaptic plasticity profiles? Does a specific range of physiological sub‐ or suprathreshold properties determine the synaptic plasticity measurements or are they independent of each other? Does the synaptic permeability parameter play any role in defining the relationship between these intrinsic and synaptic plasticity measurements? To address these questions, we plotted intrinsic measurements against measurements related to the synaptic plasticity profiles for all the 126 GC models, for two different values of baseline synaptic strength (Figure 3). We employed five intrinsic measurements (Figure 1), namely input resistance (*R*_in_), temporal summation ($S_{\alpha }$), sag ratio, maximum impedance amplitude (|*Z*|_max_), and firing rate at 150 pA (*f*_150_). Three measurements related to the synaptic plasticity profile were employed, namely percentage weight changes at $f_{i}$ = 1 Hz ($\Delta w_{1}$) and 10 Hz ($\Delta w_{10}$), modification threshold ($\theta_{m}$). We first plotted the pairwise scatter plots between the intrinsic and the synaptic plasticity measurements spanning all 126 GC models and calculated the Pearson's correlation coefficient for these pairwise scatter plots (Figure 3). We found weak pairwise correlation coefficients (−0.4 < *R* < 0.4) across all the pairs, for synaptic plasticity measurements computed with two different values of baseline synaptic strength (Figure 3a,b). These results suggest that intrinsic excitability and temporal summation are not sufficiently strong to impose specific plasticity profiles on model synapses across the heterogeneous population of models, and that several other mechanisms govern the emergence of these plasticity profiles.

### 3.4 Plasticity degeneracy: Synergistic interactions between neuronal intrinsic properties and synaptic strength result in the emergence of similar synaptic plasticity profiles

The analyses thus far assumed the baseline synaptic strength (defined by receptor densities prior to plasticity induction) to be uniform across all valid GC models. Could synapses across these neuronal models manifest similar plasticity profiles despite the expression of pronounced heterogeneities in their intrinsic properties? As baseline synaptic strength is a known modulator of plasticity profiles (Anirudhan & Narayanan, 2015; Narayanan & Johnston, 2010; Shouval et al., 2002) could a model‐dependent baseline synaptic strength allow for the emergence of similar plasticity profiles across all models?

**FIGURE 4:**
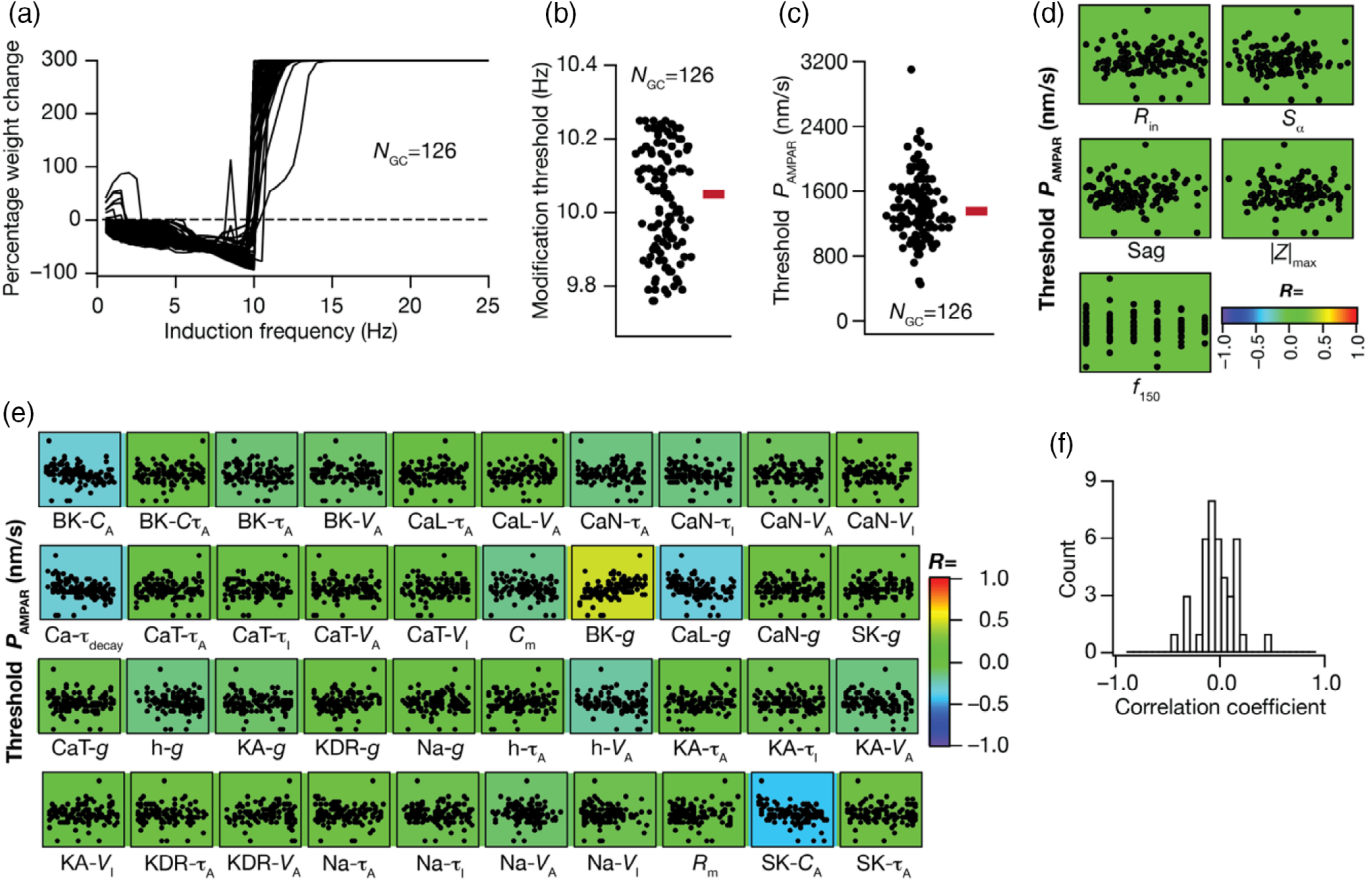
Degeneracy in the emergence of BCM‐like synaptic plasticity profile resultant from synergistic interactions between heterogeneities in intrinsic and synaptic properties. (a) Similar plasticity profiles with their modification thresholds at ~10 Hz (9.75–10.25 Hz) were obtained for all 126 granule cells (GC) models by adjusting the synaptic strength ${\bar{P}}_{\text{AMPAR}}$ for each model. (b) Beeswarm plot shows the distribution of modification threshold around 10 Hz obtained for all the GC models. (c) Beeswarm plot representing distribution of α‐amino‐3‐hydroxy‐5‐methyl‐4‐isoxazolepropionic acid receptor (AMPAR) permeability (${\bar{P}}_{\text{AMPAR}}$) values (range: 450–3100 nm/s) required to obtain similar plasticity profile (panels a,b) across the 126 GC models. (d) Pairwise scatter plots between AMPAR permeabilities shown in panel c and five intrinsic measurements, overlaid on respective color‐coded correlation values showing weak pairwise correlations, plotted for the 126 GC models. (e) Pairwise scatter plots between AMPA permeability values shown in panel c and 40 different intrinsic channel parameters, overlaid on corresponding color‐coded correlation values representing weak pairwise correlations, plotted for the 126 GC models. (f) Histogram representing the distribution of correlation coefficient values depicted in panel e.

To address these questions, we first executed an algorithm, independently for each of the 126 models, that identified the value of baseline synaptic strength (${\bar{P}}_{\text{AMPAR}}$) that yielded a synaptic plasticity profile with the modification threshold around 10 Hz ($9.75\leq \theta_{m}\leq 10.25$). Despite the considerable heterogeneities in intrinsic properties, we found that altering ${\bar{P}}_{\text{AMPAR}}$ was sufficient to achieve similar synaptic plasticity profiles across all 126 models (Figure 4a) with $\theta_{m}$ falling within the tight bound (Figure 4b). The considerable heterogeneities in intrinsic properties, however, manifested as heterogeneity in the ${\bar{P}}_{\text{AMPAR}}$ value required to achieve similar plasticity profiles. The value of ${\bar{P}}_{\text{AMPAR}}$ required to achieve similar plasticity profiles (referred to as threshold ${\bar{P}}_{\text{AMPAR}}$) spanned a wide range (Figure 4c), with the heterogeneity almost spanning an order of magnitude across models (450–3100 nm/s). Thus, although changes in ${\bar{P}}_{\text{AMPAR}}$ resulted in changes to the plasticity profile across models (Figure 2f), *specific co‐expression* of heterogeneities in synaptic (Figure 4c) and intrinsic (Figure 1) properties could result in similar plasticity profiles (Figure 4a,b).

Did the emergence of similar plasticity profiles require strong constraints on the relationship between synaptic strength and intrinsic excitability of the models? Were there strong relationships between synaptic strength and any of the biophysical parameters that defined the models that yielded similar synaptic plasticity profiles? To address these, we first computed pairwise correlation coefficients between the ${\bar{P}}_{\text{AMPAR}}$ value that was required to obtain similar plasticity profiles (from Figure 4c) and five intrinsic measurements of the respective models (from Figure 1) and found them to be weakly correlated (Figure 4d; −0.03 < *R* < 0.02). We next plotted pair wise scatter plot matrix between these ${\bar{P}}_{\text{AMPAR}}$ values (from Figure 4c) and the 40 different intrinsic parameters that defined these 126 models to explore possible parametric dependencies (Figure 4e). We found these pairwise correlation coefficients to be weak (−0.5 < *R* < 0.5; Figure 4f), with the relatively high correlation values ($\left|R\right|\approx 0.5$) spanning the relationships between ${\bar{P}}_{\text{AMPAR}}$ values and parameters governing cytosolic calcium dynamics (conductances of *L*‐type calcium and BK channels; parameters governing calcium‐dependent activation of BK and SK channels, the decay time constant of cytosolic calcium). Together, these results demonstrate that neither the intrinsic properties (spanning sub‐ and supra‐threshold intrinsic excitability and temporal summation) nor biophysical parameters (spanning passive and active properties) were sufficient to impose strong constraints on the synaptic strength required for obtaining similar plasticity profiles. These results also imply that heterogeneity‐induced variation in any parameter is compensated by *synergistic interactions spanning* several other parameters (rather than recruiting strong pairwise compensations) toward achieving plasticity profile homeostasis. Importantly, these observations clearly demonstrate that disparate parametric combinations could yield similar plasticity profiles, pointing to the expression of degeneracy in the emergence of BCM‐like synaptic plasticity profiles in DG GCs.

### 3.5 Heterogeneities and degeneracy in synaptic plasticity induced by TBS in the heterogeneous granule cell population

**FIGURE 5:**
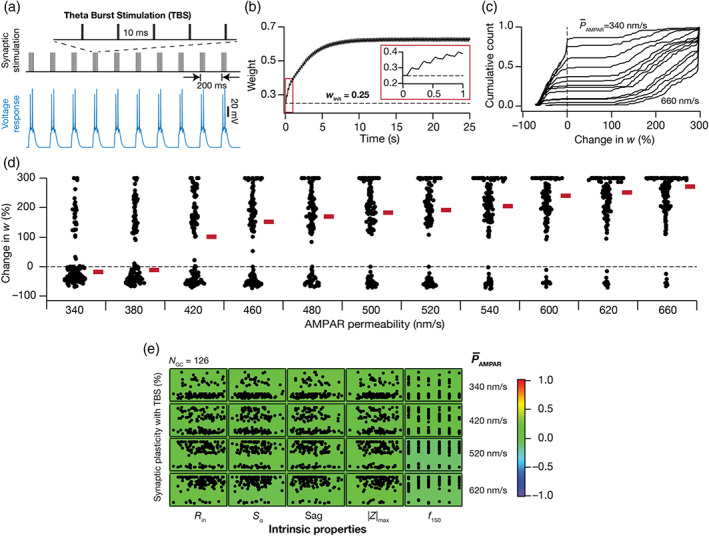
Intrinsic heterogeneities in the granule cell (GC) population translates to heterogeneities in plasticity induced by the theta burst stimulation (TBS) protocol, when synaptic properties were fixed across models. (a) Top, schematic of TBS protocol to induce synaptic plasticity. The protocol consists of bursts of stimuli with the inter burst interval set at 200 ms and each burst comprised five events separated by 10 ms interval (expanded view). *Bottom*, typical voltage response of an example GC model to TBS. (b) Plot representing evolution of α‐amino‐3‐hydroxy‐5‐methyl‐4‐isoxazolepropionic acid (AMPA) receptor weight reaching an average steady‐state value of 0.62 from initial weight value set to 0.25 as a function of time in response to TBS. *Inset*, plot showing the initial portion of the weight evolution in response to five bursts of the TBS protocol. (c,d) Cumulative histogram (c) and beeswarm plots (d) showing the amount of LTP across all models with different AMPAR permeabilities, ranging from 340 to 660 nm/s. It may be noted that number of GC models undergoing LTP increases as a function of AMPAR permeability values. (e) Pairwise scatter plots between TBS‐induced change in synaptic strength and five different intrinsic properties of all GC models, plotted for different values of baseline AMPAR permeabilities. The plots are overlaid on the respective color‐coded correlation coefficients values, which show weak correlations across all plots.

The results thus far demonstrated that while heterogeneities in intrinsic and synaptic properties could independently translate to plasticity profile heterogeneity, they could also synergistically interact to elicit similar plasticity profiles despite widespread heterogeneities in each underlying parameter. However, these observations were limited to the BCM‐like synaptic plasticity profile. To understand the dependence and robustness of these conclusions on the type of induction protocol, we turned to a more physiologically relevant and well‐established synaptic plasticity induction protocol: TBS (Figure 5a). Synapses were provided TBS, and the consequent change in synaptic weight following the calcium‐dependent dynamics (Equation 11) was computed as the difference of steady‐state weight value from its initial weight ($w_{\text{init}}$ = 0.25). The temporal evolution of synaptic weight in response to TBS (in a representative model) shows synaptic potentiation when steady‐state weight value was achieved (Figure 5b). We measured plasticity induction consequent to TBS with different values of baseline synaptic strength (${\bar{P}}_{\text{AMPAR}}$) across each of the 126 intrinsically heterogeneous models (Figure 5d).

First, for any value of ${\bar{P}}_{\text{AMPAR}}$, we observed pronounced heterogeneity in the magnitude and strength of TBS‐induced synaptic plasticity (Figure 5c,d). While synapses on certain models manifested potentiation, others showed depression for identical synapses receiving identical patterns of stimulation across models. Second, for lower values of ${\bar{P}}_{\text{AMPAR}}$ several models showed synaptic depression, whereas with higher ${\bar{P}}_{\text{AMPAR}}$, a majority manifested potentiation (Figure 5c,d). Finally, there were no strong pairwise correlations between TBS‐induced synaptic plasticity and any of the several intrinsic properties of the model neurons (Figure 5e). These results demonstrate that synaptic and intrinsic heterogeneities could independently alter the direction and the strength of TBS‐induced synaptic plasticity, without strong pairwise correlations between synaptic plasticity and neuronal intrinsic properties.

**FIGURE 6:**
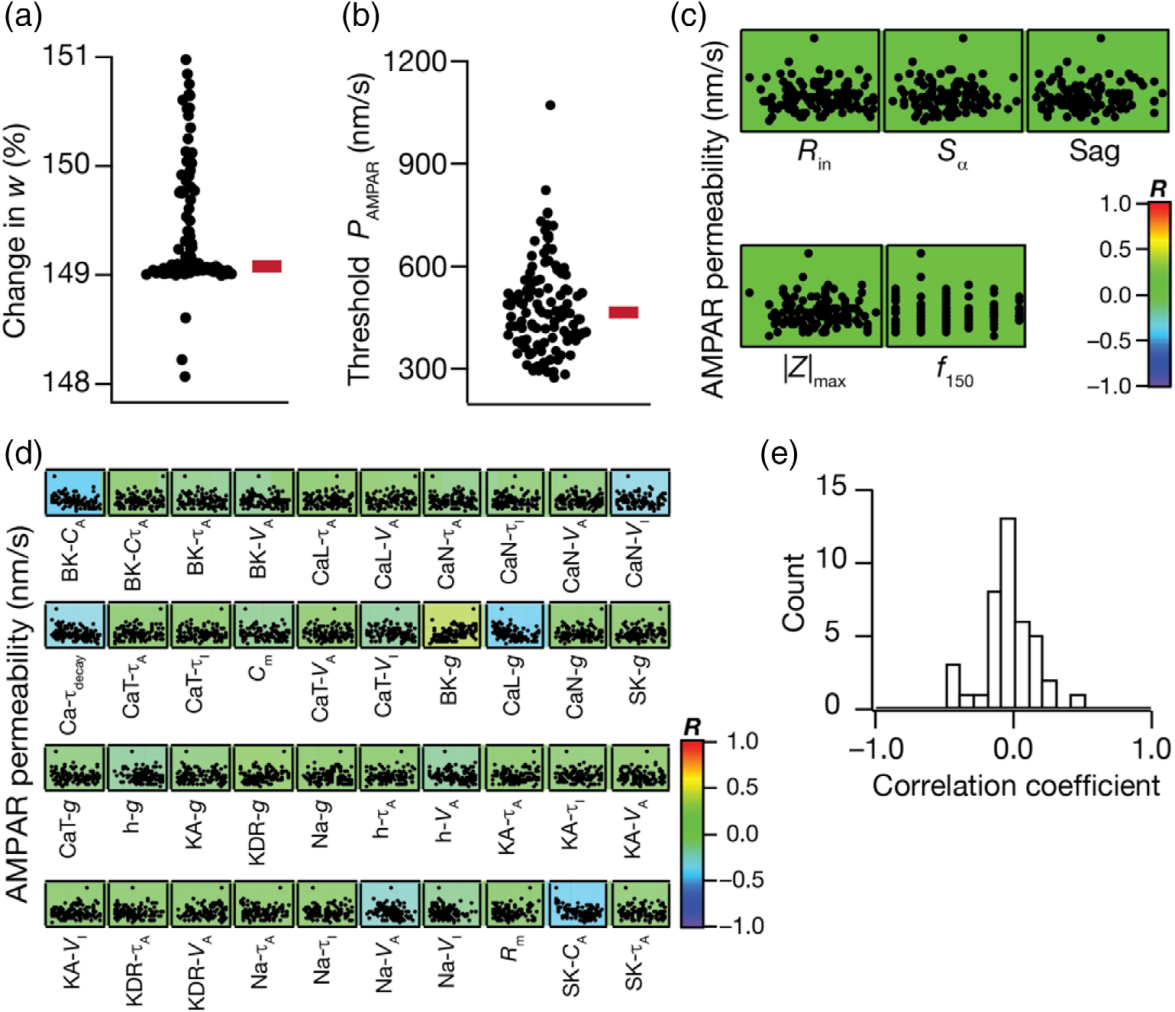
Degeneracy in eliciting the same amount of theta burst stimulation (TBS)‐induced LTP emerges from synergistic interactions between heterogeneities in intrinsic and synaptic properties. (a) Plot showing that the same amount of LTP (~150%) is obtained in different granule cell (GC) models by adjusting the baseline α‐amino‐3‐hydroxy‐5‐methyl‐4‐isoxazolepropionic acid receptor (AMPAR) permeability value. (b) Beeswarm plot representing the range of AMPAR permeabilities required to obtain ~150% LTP shown in (a). (c) Pairwise scatter plots between permeability parameter in (b) and five intrinsic measurements of the respective models, overlaid on the respective color‐coded correlation coefficients. Weak correlation values (−0.05 < *R* < 0.06) indicate the absence of pairwise dependency between the synaptic parameter and intrinsic measurements in the emergence of degeneracy. (d) Pairwise scatter plots between permeability parameter in (b) and intrinsic parameters spanning all GC models. Overlaid are respective color‐coded correlation values. (e) Histogram of correlation coefficients represented in (d). Weak correlation values (−0.4 < *R* < 0.5) indicate lack of pairwise dependency between intrinsic and synaptic parameters in the emergence of plasticity degeneracy.

We next asked whether we could tune each of these intrinsically heterogeneous populations of GC models to elicit similar amount of TBS‐induced synaptic potentiation, despite the expression of heterogeneities. To do this, for each model, we independently executed an algorithm that searched for a ${\bar{P}}_{\text{AMPAR}}$ value that resulted in ~150% TBS‐induced change in synaptic strength. We found that similar amount of LTP could be obtained across all 126 intrinsically heterogeneous models (Figure 6a). The ${\bar{P}}_{\text{AMPAR}}$ value required for yielding similar LTP, however, was heterogeneous and spanned a wide range across the 126 models (Figure 6b). Thus, the specific expression of intrinsic and synaptic heterogeneities could yield similar TBS‐induced LTP (Figure 6a,b), despite them being independently capable of altering TBS‐induced synaptic plasticity (Figure 5). Across models, none of the five intrinsic measurements (Figure 6c) or the 40 intrinsic parameters that governed the models (Figure 6d,e) manifested strong pairwise correlations with the ${\bar{P}}_{\text{AMPAR}}$ required for eliciting similar TBS‐induced LTP. There were some parameters, especially those governing calcium dynamics, that manifested relatively high values of correlation coefficients with the ${\bar{P}}_{\text{AMPAR}}$ values ($\left|R\right|\approx 0.5$), but none of them showed strong correlations. Together, these results demonstrated the expression of degeneracy in achieving similar TBS‐induced synaptic plasticity and emphasized that intrinsic properties do not impose strong constraints on synaptic parameters toward induction of similar synaptic plasticity.

### 3.6 Neurogenesis‐induced age‐dependent structural heterogeneity regulates the heterogeneity in plasticity profiles across intrinsically variable GC models

**FIGURE 7:**
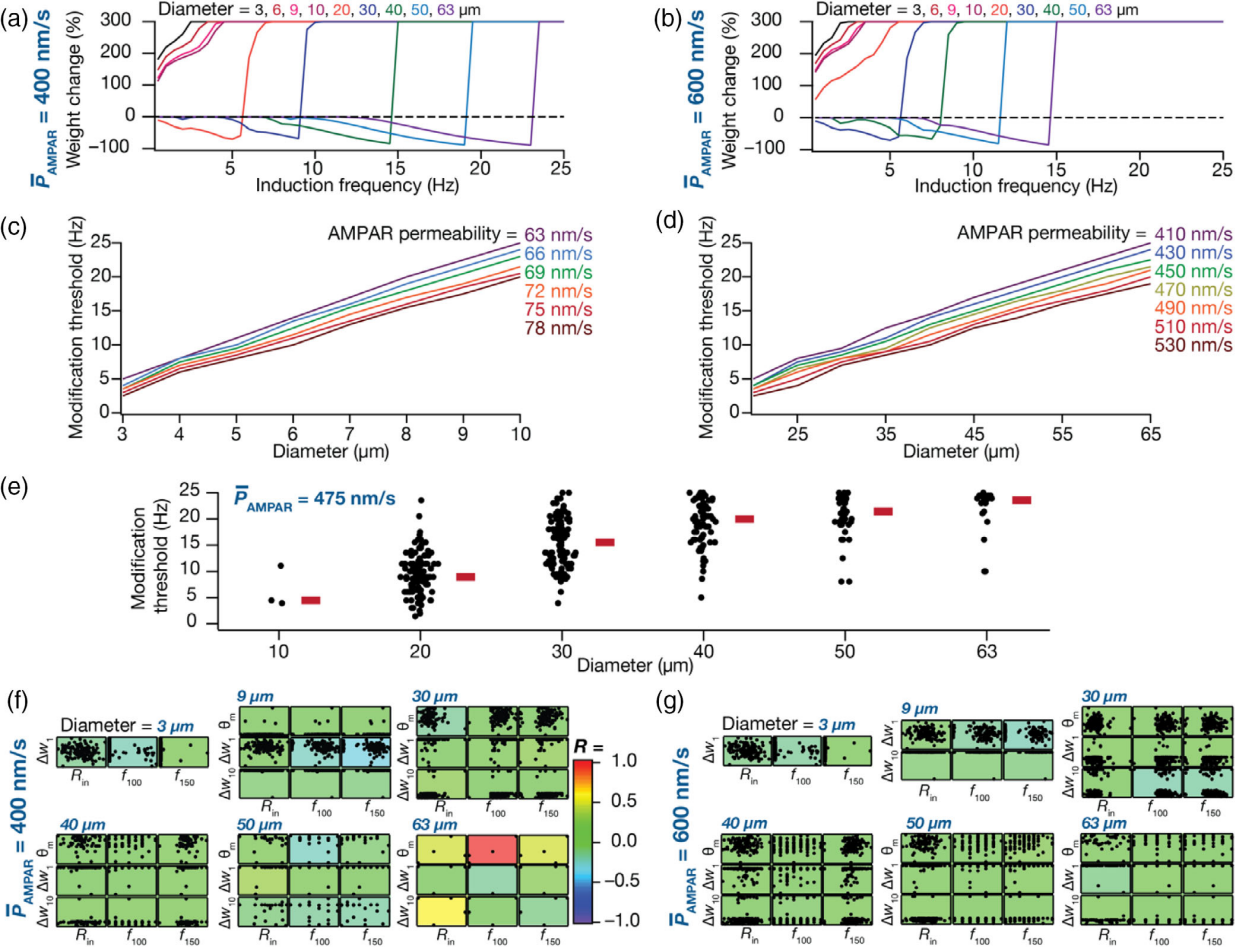
Age‐dependent structural heterogeneity in granule cell (GC) models manifests as heterogeneity in the plasticity profiles obtained from the 900 pulses protocol. (a,b) Plasticity profiles obtained with the 900‐pulses protocols with different induction frequencies, corresponding to a single GC model with different diameters for two different values of baseline α‐amino‐3‐hydroxy‐5‐methyl‐4‐isoxazolepropionic acid receptor (AMPAR) permeability: ${\bar{P}}_{\text{AMPAR}}$ = 400 nm/s (a) and ${\bar{P}}_{\text{AMPAR}}$= 600 nm/s (b). (c,d) Plots of modification threshold as functions of diameter for different values of ${\bar{P}}_{\text{AMPAR}}$ in a single GC model. Shown are plots for immature (c; 2–10 μm) and mature (d; 40–65 μm) ranges of diameters. The leftward shifts in plasticity profile observed with decreases in diameter or increases in permeability signifies lower threshold for LTP induction in the same GC model with lower diameter or higher permeability. Immature GC models undergo LTP at lower ${\bar{P}}_{\text{AMPAR}}$ values (compare permeability ranges in panel c vs. panel d) due to their highly excitable nature. (e) Beeswarm plots showing the distribution of modification threshold as a function of diameters across all GC models for a fixed ${\bar{P}}_{\text{AMPAR}}$ value of 475 nm/s. Note that the modification threshold did not fall within the tested range of induction frequencies for different models at different diameter values, thus resulting in different number of points for each diameter value (*N* = 3, 117, 121, 93, 47, and 21 from the left to right). (f) Pairwise scatter plots between different plasticity measurements: Modification threshold ($\theta_{m}$), percentage weight change at 1 Hz ($\Delta w_{1}$) and 10 Hz ($\Delta w_{10}$) and measurements of intrinsic excitability: *R*_in_, *f*_100_, and *f*_150_ for two AMPAR permeability values: ${\bar{P}}_{\text{AMPAR}}$ = 400 nm/s (f) and 600 nm/s (g), across six diameters values (3, 9, 30, 40, 50, and 63 μm). The scatter plots are overlaid to corresponding color‐coded pairwise correlation coefficients representing weak pairwise correlations across diameters and permeability values. Note that $\theta_{m}$ did not fall within the tested range of induction frequencies for different models with different diameter values, thus resulting in lesser points for certain diameter values. The axes ranges for each measurement span the entire range of the respective measurements and are different across different plots.

The DG is endowed with adult‐neurogenesis, where it takes them 4–8 weeks to fully mature and become physiologically and morphologically similar to the developmentally born neurons. Immature adult‐born GCs have reduced dendritic arborization and are highly excitable in nature with lower threshold for induction of synaptic plasticity (Aimone et al., 2014; Dieni et al., 2013; Ge et al., 2007; Huckleberry & Shansky, 2021; Schmidt‐Hieber et al., 2004). To incorporate the structural heterogeneity introduced by adult neurogenesis, we independently changed the diameter (range from 1 to 65 μm) of 126 valid mature GCs to reflect the maturation process: 2–9 μm diameter for the immature neuronal population matching the high input resistance found from electrophysiological studies (Heigele et al., 2016; Li et al., 2017; Lodge & Bischofberger, 2019; Overstreet‐Wadiche, Bensen, & Westbrook, 2006a; Overstreet‐Wadiche, Bromberg, et al., 2006; Pedroni et al., 2014; Schmidt‐Hieber et al., 2004); diameters in the 60–65 μm range formed a fully mature population, based on the diameter of the base model population set at 63 μm; and intermediate diameters range 10–60 μm resulted in an age‐dependent population at different maturation phases. To understand and quantify the dependence of plasticity profile on neurogenesis induced age‐dependent structural heterogeneity, we first employed BCM‐like 900 pulses protocol of different frequency range (0.5–25 Hz) to induce synaptic plasticity in these models. Specifically, the impact of plasticity induction was assessed in the 126 *intrinsically heterogeneous* models, with the diameter changes spanning 3–65 μm, which incorporated an additional layer of *structural heterogeneity* into each of these models. A third layer of *synaptic heterogeneity* was introduced by varying the baseline AMPAR permeability ${\bar{P}}_{\text{AMPAR}}$ value, together providing us an experimental design that allowed us to assess the impact of all the three prominent neural‐circuit heterogeneities on the synaptic plasticity profile (Figure 7a–e).

Considering an example of a single granule cell model, we found that altering the diameter of the neuron had a dramatic impact on the synaptic plasticity profile even when ${\bar{P}}_{\text{AMPAR}}$ was set at a fixed value (Figure 7a,b). Thus, in the absence of synaptic or intrinsic heterogeneities, structural changes were independently capable of altering synaptic plasticity profiles, introducing a leftward shift in the plasticity profile with reduction in the diameter (Figure 7a–d). In assessing the impact of synaptic heterogeneities, we plotted modification threshold as a function of diameter for different values of ${\bar{P}}_{\text{AMPAR}}$ and found the diameter‐dependent changes were observed across ${\bar{P}}_{\text{AMPAR}}$ values irrespective of whether the diameter was varied over immature (Figure 7c) or mature (Figure 7d) ranges. As a consequence of leftward shifts in the plasticity profile induced by reductions in diameter, the ${\bar{P}}_{\text{AMPAR}}$ value required for achieving similar modification thresholds was lower in immature neurons (Figure 7c) compared to their developing/mature counterparts (Figure 7d). However, irrespective of the ranges of diameters, increase in ${\bar{P}}_{\text{AMPAR}}$ values resulted in an expected leftward shift in the plasticity profiles (Figure 7c,d).

To address the impact of intrinsic heterogeneity on the modification threshold in the context of structural heterogeneity, we chose a specific value of ${\bar{P}}_{\text{AMPAR}}$ (475 nm/s; Figure 7e) such that there were at least some models with modification threshold value within the tested range of induction frequencies (0.5–25 Hz) across different range of diameter (between 10 and 63 μm). This was essential because reduction in diameter led to large leftward shifts in the plasticity profile. Such large shifts yielded a scenario where none of the tested induction frequencies resulted in depression thereby rendering the modification threshold to be indeterminate (e.g., diameters 3–10 μm in Figure 7a). For a fixed value of ${\bar{P}}_{\text{AMPAR}}$, we found that the modification threshold increased as a function of diameter, albeit manifesting considerable heterogeneity in the modification threshold for a given diameter value across different models (Figure 7e). For several models with diameters of 10, 40, 50, and 63 μm, the modification threshold (with ${\bar{P}}_{\text{AMPAR}}$ = 475 nm/s) was not within the tested range of induction frequencies (0.5–25 Hz), thus resulting in lesser number of models for those diameters (Figure 7e).

Were there strong relationships between intrinsic and synaptic plasticity measurements across these models across different diameters and different values of ${\bar{P}}_{\text{AMPAR}}$? To answer this, we employed three intrinsic measurements ($R_{\text{in}}$, $f_{100}$ and $f_{150}$) and three measurements of synaptic plasticity ($\theta_{m}$, $\Delta w_{1},$ and $\Delta w_{10}$), each measured for six diameter values (3, 9, 30, 40, 50, and 63 μm) and two ${\bar{P}}_{\text{AMPAR}}$ values (Figure 7f,g). We computed Pearson's correlation coefficients between the intrinsic and synaptic plasticity measurements and found weak pair wise correlations between intrinsic and plasticity measurements across different diameter and permeability values. Together, these analyses demonstrated that immature cells with relatively smaller surface areas showed a lower threshold value for LTP induction, in terms of the induction frequency (Figure 7a,b,e) and the baseline synaptic strength (immature, Figure 7c vs. mature, Figure 7d). We noted that these observations matched their electrophysiological counterparts showing that immature neurons have lower threshold for plasticity induction compared to mature neurons (Aimone et al., 2014; Dieni et al., 2013; Ge et al., 2007; Schmidt‐Hieber et al., 2004).

### 3.7 Synergistic interactions between different forms of heterogeneities resulted in the emergence of plasticity degeneracy with BCM‐like plasticity profiles

At any given time‐point, the granule cell population in the DG network comprises neurons in distinct age groups, spanning the entire range of just‐born to fully mature neurons. Thus, based on our analyses so far, the consequent structural and intrinsic heterogeneities could result in distinct plasticity profiles with different ranges of modification thresholds. However, we had demonstrated earlier that similar plasticity profiles could be achieved across different intrinsically heterogeneous GC neurons, if the baseline synaptic strength was adjusted appropriately (Figure 4). Although intrinsic neural properties and synaptic strength manifested considerable heterogeneities when viewed independently, together they were able to yield very similar plasticity profiles (Figure 4). Could such plasticity degeneracy manifest even in presence of neurogenesis‐induced structural heterogeneity? Could similar plasticity profiles be achieved despite the concomitant expression of intrinsic, synaptic, and structural heterogeneities in the DG neuronal population?

**FIGURE 8:**
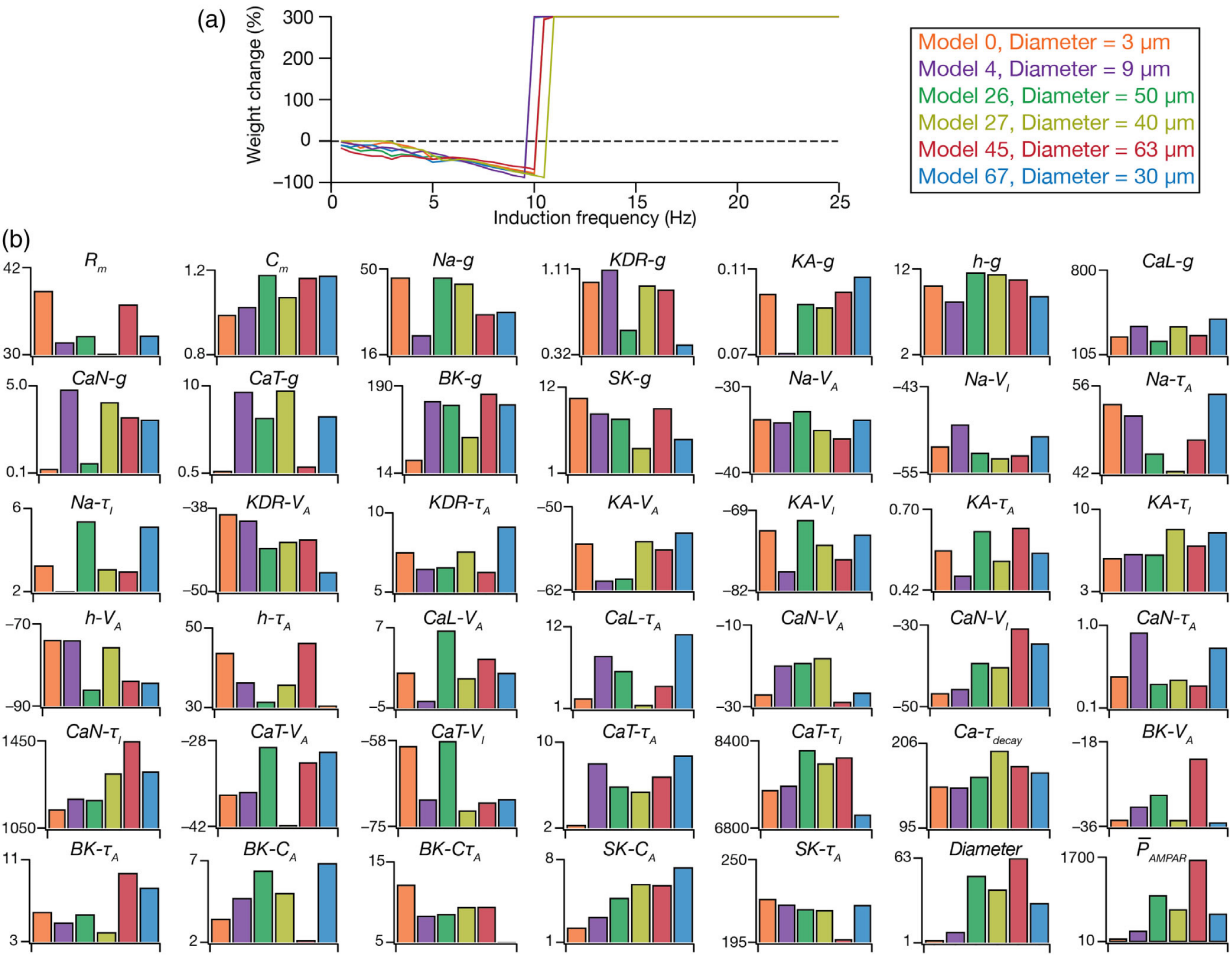
Illustration of degeneracy in the emergence of plasticity profiles spanning biophysical, structural, and synaptic parameters using six models. (a) Frequency‐dependent plasticity profiles plotted for six intrinsically disparate models with different diameters and ${\bar{P}}_{\text{AMPAR}}$ values yield similar plasticity profile with modification threshold at ~10 Hz. (b) Plots, for each of the six models shown in panel a, of the 40 intrinsic passive and active properties (listed in Table 1 with units), the diameter (in μm) and the ${\bar{P}}_{\text{AMPAR}}$ (in nm/s) values required to get the modification threshold to be ~10 Hz. The plots for each of the 40 intrinsic parameters (Table 1) and diameter (1–63 μm) span their entire search range. Note that the ranges of each parameter across the six models are highly variable (b), spanning a large portion of the parameter's search range, despite the similarity of the plasticity profiles (a)

To assess these questions, we first selected six intrinsically distinct GC models (from the population of 126 models) and assigned different values of diameters to each of these six models. We then employed an algorithm to find a synaptic permeability value (${\bar{P}}_{\text{AMPAR}}$) that yielded plasticity profiles endowed with their modification threshold at ~10 Hz with the 900‐pulse protocol (Figure 8a). We found the synaptic plasticity profiles for each of these six models, endowed with their respective ${\bar{P}}_{\text{AMPAR}}$ provided by the algorithm, to be similar across the entire range of induction frequencies (0.5–25 Hz) (Figure 8a). We then plotted each of the 42 parameters underlying these six models (40 intrinsic parameters in Table 1, diameter as the structural parameter, and ${\bar{P}}_{\text{AMPAR}}$ governing the synapse) and found each of them to span their respective ranges (Figure 8b). These analyses illustrate that models built with very different structural, intrinsic, and synaptic properties (Figure 8b) could together yield very similar synaptic plasticity profile (Figure 8a), thus demonstrating the emergence of plasticity degeneracy despite widespread variability in all underlying parameters.

**FIGURE 9:**
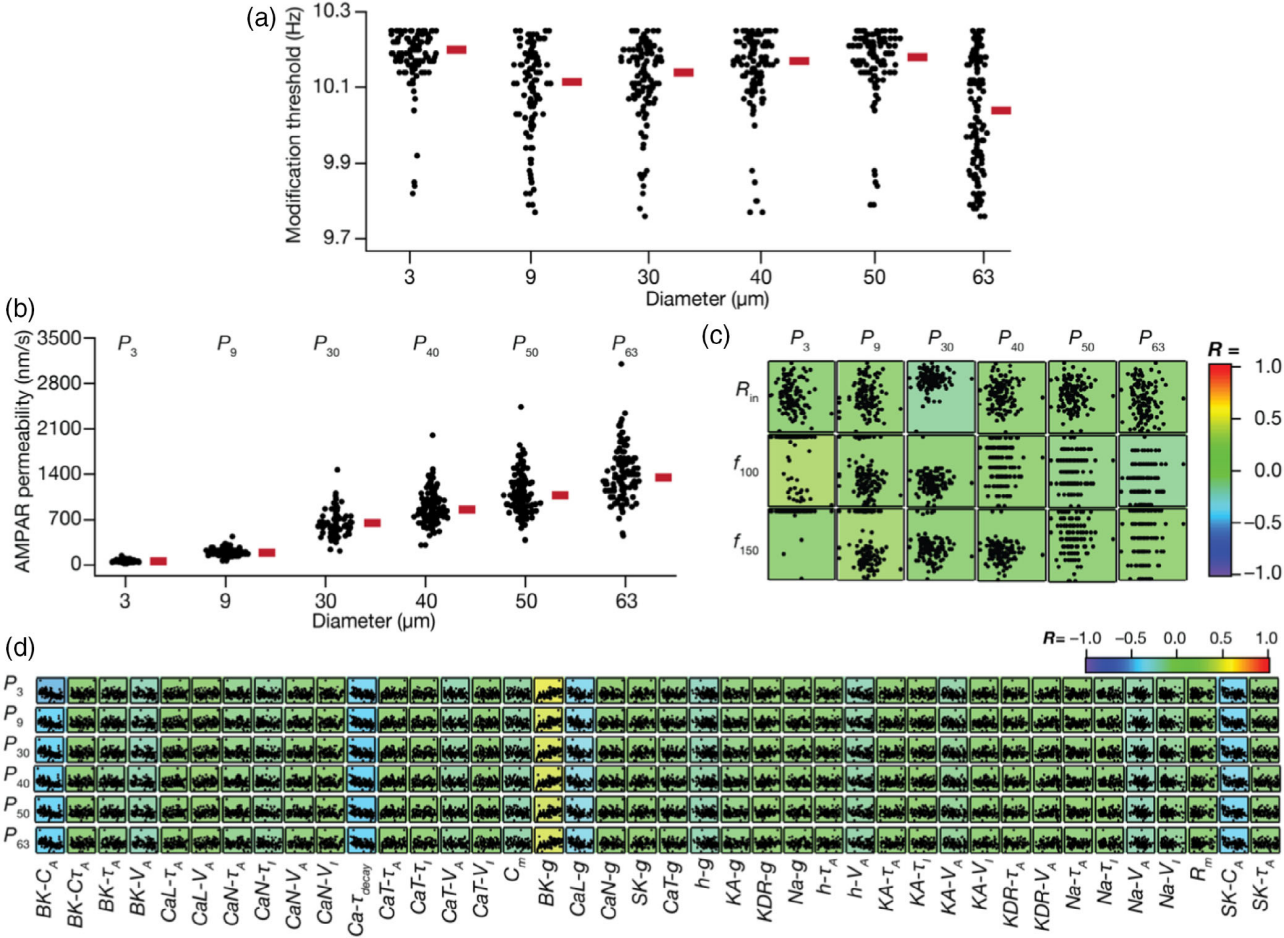
Emergence of plasticity degeneracy due to synergistic interactions between age‐dependent structural, synaptic, and intrinsic heterogeneities with weak pairwise correlations. (a) Plot representing the distribution of modification threshold for all GC models across different diameters to obtain modification threshold of ~10 Hz by adjusting α‐amino‐3‐hydroxy‐5‐methyl‐4‐isoxazolepropionic acid receptor (AMPAR) permeability for each model. (b) Plot depicting the distribution of AMPAR permeability values required to obtain plasticity profiles with modification threshold of ~10 Hz (shown in panel a), across different diameter values. (c) Pairwise scatter plots between AMPA permeability values depicted in *B* and intrinsic excitability measurements (*R*_in_, *f*_100_, and *f*_150_) across different diameters, overlaid on respective color‐coded correlation coefficient values. (d) Pairwise scatter plots showing distribution of intrinsic parameters across AMPA permeabilities that yielded ~10 Hz modification threshold across different diameters. The scatter plots are overlaid on color‐coded pairwise correlation coefficient values showing weak pairwise correlations.

We expanded the scope of our analyses to span all 126 intrinsically heterogeneous models, each spanning six diameter values (3, 9, 30, 40, 50, and 63 μm) and employed our algorithm to find a ${\bar{P}}_{\text{AMPAR}}$ that would yield a modification threshold of ~10 Hz ($9.75\leq \theta_{m}\leq 10.25$) in each of these (126×6=756) models (Figure 9a). We were able to find ${\bar{P}}_{\text{AMPAR}}$ values that yielded similar modification thresholds, with the required ${\bar{P}}_{\text{AMPAR}}$ increasing with increase in diameter (Figure 9b). We did not find strong correlations between the ${\bar{P}}_{\text{AMPAR}}$ value required for achieving similar plasticity profiles and the respective intrinsic measurements (Figure 9c). These observations rule out the requirement of strong counterbalances between intrinsic and synaptic properties, within each of the six assessed diameters. Similarly, there were no strong correlations between the threshold ${\bar{P}}_{\text{AMPAR}}$ value and each of the 40 intrinsic parameters (Table 1), for models with each of the six diameter values (Figure 9d). Together, these results demonstrated that degeneracy in the emergence of plasticity profiles is not dependent on strong pairwise compensations between synaptic properties and individual intrinsic measurements (Figure 9c) or parameters (Figure 9d). These analyses suggest a role for synergistic interactions among structural, intrinsic, and synaptic parameters in yielding *similar* plasticity profiles.

### 3.8 Synergistic interactions between synaptic, intrinsic, and structural heterogeneities governed TBS‐induced synaptic plasticity

**FIGURE 10:**
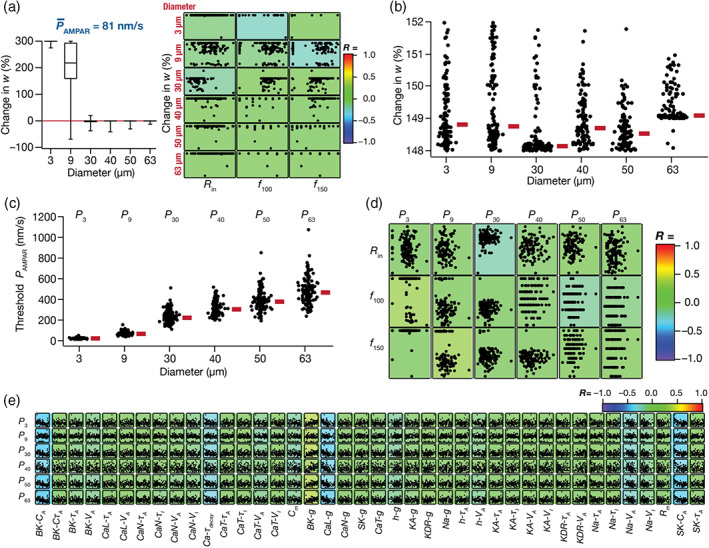
Heterogeneities and degeneracy in synaptic plasticity achieved with theta burst stimulation (TBS) protocol in models endowed with age‐dependent structural, synaptic, and intrinsic heterogeneities. (a) Left, age‐dependent structural heterogeneity in the population of GC models translated to heterogeneity in the amount of plasticity achieved with tTBS protocol when baseline synaptic strength was fixed to 81 nm/s. Shown is the amount of plasticity achieved for models in the intrinsically heterogeneous model population, with the diameter altered to assess the impact of structural heterogeneities. Right, pairwise scatter plots between different plasticity measurements associated with TBS versus measurements of intrinsic excitability: *R*_in_, *f*_100_, and *f*_150_ for a fixed value of baseline synaptic strength, ${\bar{P}}_{\text{AMPAR}}$ = 81 nm/s, across six diameter values (3, 9, 30, 40, 50, and 63 μm). The scatter plots are overlaid to corresponding color‐coded pairwise correlation coefficients representing weak pairwise correlations across diameters and permeability values. The axes ranges for each measurement span the entire range of the respective measurements and are different across different plots. (b–e) Degeneracy in eliciting the same amount of TBS‐induced LTP emerges from synergistic interactions between heterogeneities in structural, intrinsic, and synaptic properties. (b) Plot showing that the same amount of LTP (~150%) is obtained in different GC models, across six different diameter values to assess the impact of structural heterogeneities, by adjusting ${\bar{P}}_{\text{AMPAR}}$. (c) Beeswarm plot representing the range of AMPAR permeabilities required to obtain ~150% LTP shown in b, for each of the six diameter values. (d) Pairwise scatter plots between permeability parameter in c and three intrinsic measurements of the respective models, overlaid on the corresponding color‐coded correlation coefficients. Plots are shown for each of the six diameter values. (e) Pairwise scatter plots between permeability parameter in b and intrinsic parameters spanning all GC models. Overlaid are respective color‐coded correlation values.

We repeated our analyses on the impact of the three forms of heterogeneities with the TBS protocol. First, we found that heterogeneities in structural properties could alter the amount of synaptic plasticity achieved with TBS across the intrinsically heterogeneous model population, when structural heterogeneity was introduced by altering diameters to six different values representative of immature and mature granule cell populations. For these analyses, we fixed the ${\bar{P}}_{\text{AMPAR}}$ value and found that the amount of plasticity obtained reduced with increasing value of diameter (Figure 10a, left), thus demonstrating a lower threshold on ${\bar{P}}_{\text{AMPAR}}$ for inducing LTP in immature neurons. We also found that there was no correlation between the amount of plasticity achieved and the respective intrinsic properties, for each value of diameter assessed (Figure 10a, right). Second, to explore plasticity degeneracy with the TBS protocol, we next found ${\bar{P}}_{\text{AMPAR}}$ values that yielded similar levels of synaptic plasticity of ~150% (148%–152%) for each of the 126 intrinsically heterogeneous models, with six different values of diameters (Figure 10b,c). The ${\bar{P}}_{\text{AMPAR}}$ value required for achieving similar plasticity increased with increase in diameter and did not manifest strong correlations with either the respective intrinsic measurements (Figure 10d) or the intrinsic parameters (Figure 10e) for each value of the diameter.

Together, our analyses demonstrated that each of intrinsic (Figures 2d–f, 5d, 7e, and 10a), synaptic (Figures 2d–f, 5c,d, and 7c,d), and structural (Figures 7e and 10a) heterogeneities could independently introduce heterogeneities in the plasticity profiles, irrespective of the protocol employed. However, when they coexpress, these disparate forms of heterogeneities could synergistically interact with each other to yield very similar plasticity profiles (Figures 4a–c, 6a,b, 8, 9 and 10b,c), irrespective of the induction protocol employed. Across our analyses spanning different plasticity protocols, assessing heterogeneities or degeneracy in plasticity profiles, we did not find strong correlations between synaptic properties plotted against intrinsic measurements (Figures 3, 4, 5e, 6c, 7f,g, 9c, and 10d) or intrinsic parameters (Figures 4e, 6d, 9d, and 10e) of the model populations. These results suggested that the measurements of intrinsic excitability and temporal summation are not sufficiently strong to impose specific synaptic plasticity profiles.

### 3.9 Importance of adult neurogenesis‐induced structural heterogeneities in lowering plasticity induction threshold and recruiting engram cells based on intrinsic excitability

In our analyses thus far, we noted that intrinsic excitability parameters were not strong enough to constrain synaptic plasticity induction, with a consistent lack of strong correlations between synaptic plasticity measurements and intrinsic excitability (Figures 4, 5, 6, 7, 9, and 10). This is in contrast with the literature where a critical role for intrinsic excitability has been postulated in reducing the threshold for plasticity induction and in individual neurons being recruited as engram cells for a new context (Ge et al., 2007; Huckleberry & Shansky, 2021; Josselyn & Frankland, 2018; Josselyn & Tonegawa, 2020; Lau et al., 2020; Lodge & Bischofberger, 2019; Park et al., 2016; Pignatelli et al., 2019; Schmidt‐Hieber et al., 2004; Yiu et al., 2014). How do we reconcile these observations? Thus far in our correlation analyses, we have focused *independently* on mature versus immature populations, treating analyses within each diameter to be independent of others (Figures 7, 9, and 10). However, in physiological scenarios where there is coexistence of cells of different ages, it is important to ask whether immature cells have an *advantage* over their mature counterparts in manifesting lower threshold for plasticity and thereby being recruited by afferent inputs. It is therefore essential that the analyses *span all ages of cells* rather than treating them as independent populations.

**FIGURE 11:**
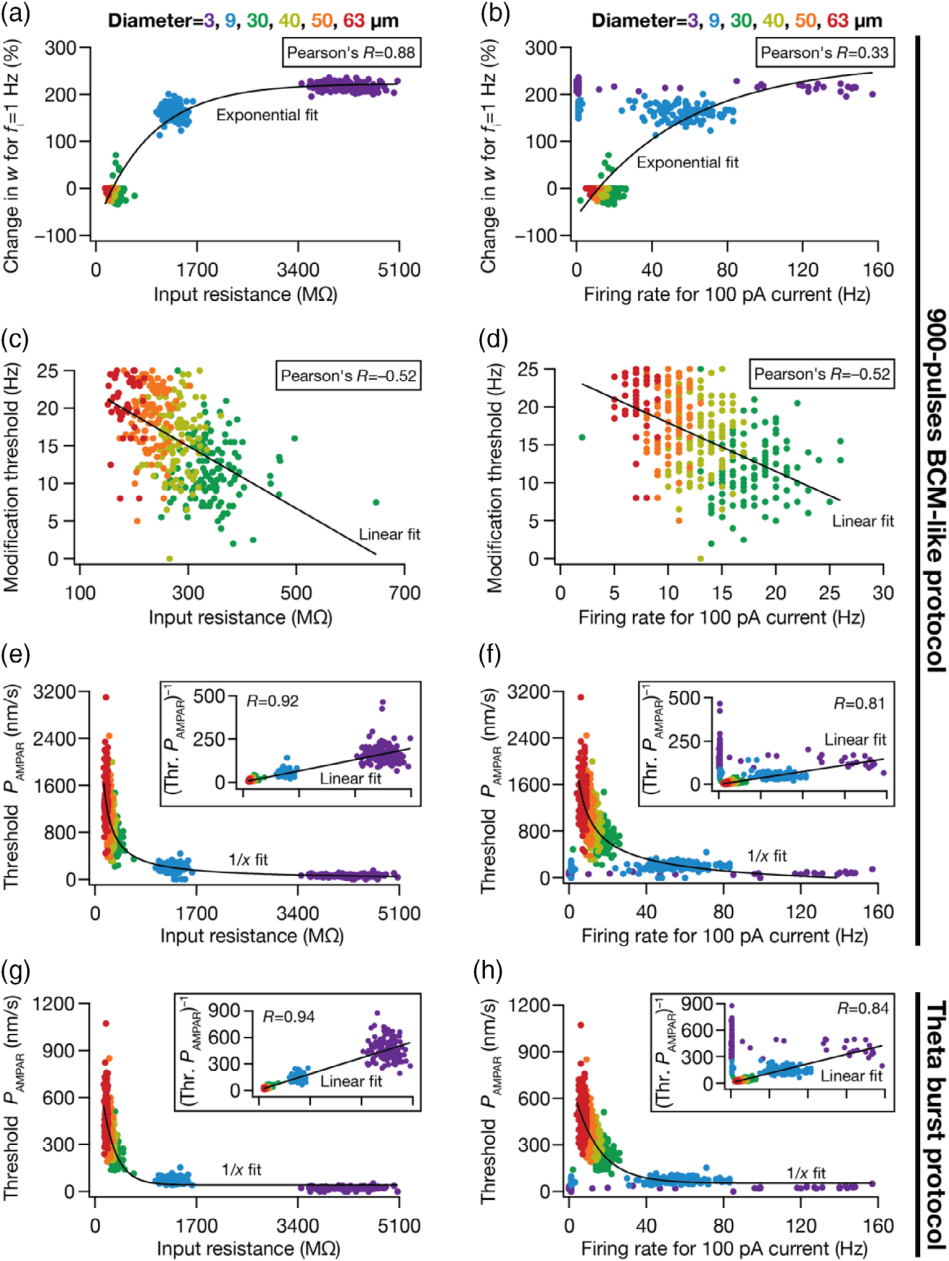
The dominant role of structural heterogeneities in regulating plasticity profiles with the BCM‐like and theta burst stimulation (TBS) plasticity protocols. (a,b) Percentage weight change at 1 Hz ($\Delta w_{1}$) with the 900‐pulses protocol plotted against input resistance (a) and firing rate for 100 pA current injection (b) for models in the intrinsically heterogeneous population, with each model assessed at six different diameter values (3, 9, 30, 40, 50, and 63 μm). (c,d) Same as (a,b), plotted for modification threshold ($\theta_{m}$) on the *y* axis. For panels (a–d), the data from Figure 7g (${\bar{P}}_{\text{AMPAR}}$ = 600 nm/s) are plotted together for all diameters. (e,f) Same as (a,b), plotted for the AMPAR permeabilities required to achieve a modification threshold of ~10 Hz (with the 900‐pulses protocol), referred to as threshold $P_{\text{AMPAR}}$, on the *y* axis. For panels (e,f), the data from Figure 9c are plotted together for all diameters. The insets in panels (e) and (f) depict the inverse of threshold $P_{\text{AMPAR}}$ plotted against $R_{\text{in}}$ or $f_{100}$, respectively, to illustrate the 1/*x* relationship between threshold $P_{\text{AMPAR}}$ versus $R_{\text{in}}\;$ and threshold $P_{\text{AMPAR}}$ versus $f_{100}$. (g,h) Same as (e,f), but plotted threshold $P_{\text{AMPAR}}$ was computed to achieve ~150% synaptic plasticity with the TBS protocol. For panels (g,h), the data from Figure 10d are plotted together for all diameters.

Therefore, we plotted the amount of plasticity induced by the 900‐pulses protocol at $f_{i}$ = 1 Hz ($∆w_{1}$; Figure 11a,b) and the modification threshold obtained with the 900‐pulses protocol ($\theta_{m}$; Figure 11c,d), computed with a fixed value of ${\bar{P}}_{\text{AMPAR}}$, against respective intrinsic excitability measurements ($R_{\text{in}}$ and $f_{100}$) spanning *all diameter values* across all neurons in the intrinsically heterogeneous population. We found strong relationships of synaptic plasticity measurements with measurements of intrinsic excitability (Figure 11a–d). Specifically, our analyses showed that the amount of induced plasticity was higher in neurons with high excitability (Figure 11a,b) and that the plasticity profile manifested a strong leftward shift with increased excitability (Figure 11c,d). Thus, while intrinsic excitability was insufficient to impose strong correlations on synaptic plasticity measurements in the absence of structural heterogeneities in the neural population (Figures 7, 9, and 10), quantitative introduction of structural heterogeneities allows the consequent intrinsic excitability changes to impose strong constraints on the synaptic plasticity profiles. In other words, although DG GCs are endowed with considerable baseline heterogeneities, the introduction of an immature population through adult neurogenesis is essential for these neurons to be specifically recruited in a new context during engram formation (Huckleberry & Shansky, 2021; Josselyn & Frankland, 2018; Josselyn & Tonegawa, 2020; Lau et al., 2020; Lodge & Bischofberger, 2019; Park et al., 2016; Pignatelli et al., 2019; Schmidt‐Hieber et al., 2004; Yiu et al., 2014).

There are lines of evidence that the synaptic strength of inputs to immature DG GCs is lower compared to their mature counterparts (Dieni et al., 2016; Li et al., 2017; Mongiat et al., 2009). Are immature neurons with such low baseline synaptic strengths capable of effectuating synaptic plasticity comparable to their mature counterparts? To address this, we first plotted the threshold $P_{\text{AMPAR}}$ required to elicit the same modification threshold (of ~10 Hz) with the 900‐pulses protocol as functions of intrinsic excitability measurements, spanning *all diameter* values across all neurons in the intrinsically heterogeneous population (Figure 11e,f). We found that in immature neurons with high excitability, even small values of ${\bar{P}}_{\text{AMPAR}}$ were sufficient to achieve synaptic plasticity profiles comparable with their mature counterparts (Figure 11e). Importantly, although threshold $P_{\text{AMPAR}}$ values from *individual* neuronal populations of different diameters did not manifest strong correlations with intrinsic measurements (Figure 9c), there was a strong inverse relationship between threshold $P_{\text{AMPAR}}$ values and $R_{\text{in}}$ (Figure 11e) as well as $f_{100}$ (Figure 11f) when neurons with all diameters were considered together. Finally, with reference to the TBS protocol, we found that small values of ${\bar{P}}_{\text{AMPAR}}$ were sufficient to achieve synaptic plasticity of ~150% in immature neurons (Figure 11g). Strong inverse relationships manifested between threshold $P_{\text{AMPAR}}$ and measurements of intrinsic excitability with the TBS protocol as well (Figure 11g,h). Together, these analyses demonstrated the essential requirement of structural heterogeneity comprising immature neuronal populations in specifically recruiting high‐excitability neuronal populations to induce synaptic plasticity, and in ensuring that sufficient plasticity is induced despite low density of synaptic receptors in immature neurons.

## 4 DISCUSSION

The principal goal of this study was to assess the mechanistic basis for the expression of plasticity heterogeneities. Plasticity heterogeneity is defined as the variability observed in the amount of plasticity induced by identical activity patterns across cells and synapses of the same subtype. We demonstrate that disparate forms of neural‐circuit heterogeneities, spanning intrinsic, synaptic, and structural properties, could provide a mechanistic substrate for the expression of plasticity heterogeneities. These neural‐circuit heterogeneities could either act individually or in unison in mediating plasticity heterogeneities. Our analyses demonstrate that structural heterogeneities, introduced by the expression of adult neurogenesis in the DG, are the dominant form of heterogeneity that drives plasticity heterogeneities. However, our analyses caution against construing the manifestation of neural‐circuit heterogeneities to be direct evidence for the expression of plasticity heterogeneities. This note of caution emanates from our demonstration of plasticity profile degeneracy, whereby similar plasticity profiles were attained across a population of models, despite pronounced heterogeneities in their synaptic, intrinsic, and structural properties.

### 4.1 Heterogeneities spawn heterogeneities: Disparate forms of biophysical and structural heterogeneities could independently drive physiologically crucial heterogeneities in plasticity profiles

We constructed multiple populations of DG granule cell models to reflect heterogeneities in neuronal passive properties, ion‐channel properties, calcium‐handling mechanisms, synaptic strength, and neural structure of DG GCs of different ages. Each of these heterogeneities was incorporated into our model populations with strong physiological constraints on multiple intrinsic properties (Table 2), thus ensuring the physiological relevance of our conclusions. We employed two well‐established synaptic plasticity protocols to demonstrate that each of intrinsic, synaptic, and structural heterogeneities independently result in heterogeneities in the amount of plasticity induced. These observations held for both plasticity protocols, one involving 900 pulses of different induction frequencies and another employing TBS. In electrophysiological experiments assessing synaptic plasticity, there is pronounced heterogeneity in the amount of plasticity induced with any induction protocol. Specifically, whereas the same protocol might elicit 300% LTP in certain neurons, in other neurons of the same subtype in the same brain region, the protocol results in 10% LTP. Such neuron‐to‐neuron and animal‐to‐animal variability in the amount of plasticity induced is typically not analyzed quantitatively, with the data typically represented using summary statistics and interpretations drawn from the average plasticity across different cells from different animals. However, given the role of such differential plasticity across different neurons in resource allocation and in engram formation, it is essential to not just report these heterogeneities but also examine the mechanisms underlying such cell‐to‐cell differences.

To emphasize the critical roles played by these plasticity heterogeneities across different cells and different synapses, let us consider an extreme scenario where these heterogeneities were absent. This would translate to all synapses across all cells undergoing the *same* amount of plasticity for any given context, together resulting in the absence of context‐dependent recruitment/allocation of synapses or cells that are critical for engram cell formation and decorrelation. From the engram cell formation perspective, there are several lines of evidence to suggest context‐dependent plasticity in a *subset* of cells that are recruited to encode a new context (Josselyn & Frankland, 2018; Josselyn & Tonegawa, 2020; Lau et al., 2020; Lodge & Bischofberger, 2019; Park et al., 2016; Pignatelli et al., 2019; Schmidt‐Hieber et al., 2004; Yiu et al., 2014). In addition, afferent connectivity has been demonstrated to be a dominant mediator of neural decorrelation (Mishra & Narayanan, 2019), with strong lines of evidence suggesting that afferent connectivity is actively driven by differences in plasticity profiles across different GCs (Aimone et al., 2006, 2009; Aimone et al., 2014; Ge et al., 2007; Li et al., 2017; Lodge & Bischofberger, 2019; Luna et al., 2019; Schmidt‐Hieber et al., 2004). Thus, in the absence of plasticity heterogeneities, the critical role of differential plasticity in mediating differential connectivity to neurons in the DG during encoding and storage process would be hampered. Our study explores the mechanistic basis for such heterogeneity and traces the potential origins to the pronounced heterogeneities in intrinsic, synaptic, and structural properties of DG GCs. These analyses emphasize the need for studies that assess neural plasticity to quantitatively report plasticity heterogeneities and to trace their origins, under physiological or pathological conditions.

### 4.2 Heterogeneities underlying degeneracy: Synergistic interactions among different forms of biophysical and structural heterogeneities could yield similar plasticity profiles

We demonstrated that even the expression of heterogeneities in all *of* structural, synaptic, and intrinsic neuronal properties does not *necessarily* have to translate to heterogeneities in synaptic plasticity profiles. Specifically, we showed that very similar plasticity profiles could be achieved with disparate combinations of neuronal passive properties, ion‐channel properties, calcium‐handling mechanisms, synaptic strength, and neural structure of DG GCs of different ages (Figures 4, 6, and 8, 9, 10). Independently observed, these properties manifested widespread heterogeneities with no pairwise relationships (Figures 4, 6, 9, and 10). But when seen together, these heterogeneities synergistically interacted with each other to achieve degeneracy in the emergence of synaptic plasticity profiles. There are computational (Beining, Mongiat, et al., 2017; Mishra & Narayanan, 2019, 2021b) and electrophysiological (Mishra & Narayanan, 2021a) lines of evidence for DG GCs to manifest ion‐channel degeneracy in the expression of their characteristic intrinsic properties. However, similarity in baseline electrophysiological properties of different neurons does not necessarily translate to similarity in terms of how these neurons react to plasticity‐inducing stimuli (Anirudhan & Narayanan, 2015; Rathour & Narayanan, 2019; Srikanth & Narayanan, 2015).

Here, we have demonstrated and expanded the scope for the expression of degeneracy in DG GCs beyond ion‐channel degeneracy and beyond achieving characteristic intrinsic properties. Specifically, we have demonstrated the manifestation of degeneracy in the emergence of plasticity profiles, independently for two different induction protocols, with the analyses *concomitantly* incorporating structural heterogeneities driven by adult‐neurogenesis, heterogeneities in intrinsic neuronal properties, and heterogeneities in synaptic strength (Figures 8, 9, 10). Importantly, this form of degeneracy was demonstrated in a heterogeneous population of neurons that manifested physiologically constrained (Table 2) neural properties, including those, which were not initially assessed (Figure 1). This population included immature cells whose excitability measurements were matched by altering their surface area. Thus, this population of neurons manifested degeneracy in the expression of physiologically matched neural intrinsic properties and showed plasticity degeneracy with the concomitant expression of all forms of neural heterogeneities. In comparing with previous studies on degeneracy, we note that these studies accounted for degeneracy either in characteristic neuronal intrinsic properties (Mishra & Narayanan, 2019, 2021a, 2021b) or in plasticity profiles (Anirudhan & Narayanan, 2015), but not together. Our study demonstrates the expression of degeneracy in the concomitant emergence of characteristic neuronal intrinsic properties and of characteristic plasticity profiles, while considering a superset of model parameters and measurements that span all ages of GCs in the DG.

The predominant implication for the expression of degeneracy in the concomitant emergence of intrinsic properties and plasticity profiles is the explosion in the degrees of freedom available for the neurons to achieve these characteristic features, thereby providing multiple routes to achieving functional robustness (Edelman & Gally, 2001; Goaillard & Marder, 2021; Rathour & Narayanan, 2019). In addition, given the expression of such degeneracy, it is essential that the theoretical and experimental analyses recognize that the mappings between structural components and functional outcomes are many‐to‐many and avoid reductionist oversimplifications of structure–function relationships (Goaillard & Marder, 2021; Mishra & Narayanan, 2021a, 2021b; Rathour & Narayanan, 2019). It is therefore critical that experimental and computational analyses explicitly account for heterogeneities in neural circuit properties and for the expression of degeneracy in the emergence of baseline neural properties and plasticity profiles. It is important to independently and systematically assess the strong interactions among different forms of heterogeneities. There could be scenarios where plasticity heterogeneities manifest in the absence of cellular‐scale heterogeneities, owing to heterogeneities in biochemical signaling cascades across cells. In addition, as shown here, there could be scenarios where heterogeneities in one property are counterbalanced by heterogeneities in others, together yielding plasticity degeneracy (Figures 4, 6 and 8, 9, 10).

### 4.3 Dominant role of structural heterogeneities in introducing plasticity heterogeneities across neurons: Selective recruitment and resource allocation during engram cell formation

A role for intrinsic excitability has been postulated in reducing the threshold for plasticity induction and in individual neurons being recruited as engram cells, toward encoding a new context (Ge et al., 2007; Josselyn & Frankland, 2018; Josselyn & Tonegawa, 2020; Lau et al., 2020; Lodge & Bischofberger, 2019; Park et al., 2016; Pignatelli et al., 2019; Schmidt‐Hieber et al., 2004; Yiu et al., 2014). However, in the individual population of mature or immature cells, we demonstrated that intrinsic excitability and temporal summation heterogeneities were insufficient to impose strong constraints on plasticity‐related measurements (Figures 3, 4, 5e, 6c, 7f,g, 9c, and 10d). However, when the entire population covering mature and immature cells were considered *together*, it was clear that there were strong relationships between intrinsic excitability and measurements associated with synaptic plasticity (Figure 11). These results highlighted the dominance of structural heterogeneities, introduced through adult neurogenesis, in introducing heterogeneities in plasticity profiles that are essential for effective execution of encoding and storage roles of the DG.

We explored a range of AMPAR strengths across neurons with different diameters and demonstrated that similar levels of synaptic plasticity could be achieved despite the low synaptic strength (Figure 11e–h) that is observed onto these immature neurons (Dieni et al., 2016; Li et al., 2017; Mongiat et al., 2009). The lower ranges of AMPAR strengths may indeed be an essential requirement for keeping the plasticity in a useful physiological range, because higher AMPAR strengths in immature neurons might result in large magnitude and unstable plasticity dynamics. Together, the expression of adult neurogenesis amplifies heterogeneities in intrinsic excitability properties and in synaptic strengths. Our study demonstrates that these amplified heterogeneities could be critical in defining plasticity heterogeneity across different neurons, and in defining a role for intrinsic excitability in recruitment/allocation of engram cells. Specifically, the heterogeneities typically seen in mature cells (with input resistance in the range of 100–300 MΩ) are not sufficient to enforce strong correlations of neural excitability measurements with the expression of synaptic plasticity. Higher input resistance values (in the GΩ range) that distinguish the immature neurons from their mature counterparts are essential to significantly lower the synaptic plasticity induction threshold and drive the recruitment of these as engram cells for new contexts.

### 4.4 Limitations and future directions

Although our model population spanned all forms of heterogeneities and was physiologically constrained in several ways, there are some limitations in the model and in parametric choices. First, the computational cost for each plasticity simulation involved either 900 pulses of different $f_{i}$ values ranging from 0.5 to 25 Hz in steps of 0.5 Hz, or TBS repeated for 150 times. Both protocols were repeated for each of the 126 intrinsically heterogeneous models with different ${\bar{P}}_{\text{AMPAR}}$ and several diameter values. To partially offset for this tremendous computational cost, we had employed a single‐compartmental model to assess the impact of neural‐circuit heterogeneities on plasticity profiles. However, it is essential that future studies account for morphological reconstructions of DG GCs with experimentally determined somato‐dendritic distributions of channels and receptors and assess plasticity profiles for synapses placed at different somato‐dendritic locations (Sjostrom & Hausser, 2006). These studies could also specifically employ immature versus mature dendritic morphologies rather than introducing surface area changes through change in diameter. Such analyses, in conjunction with electrophysiological experiments, would address questions on heterogeneities in developmentally versus adult‐born GCs (Abrous & Wojtowicz, 2015; Anacker & Hen, 2017; Beining, Jungenitz, et al., 2017; Cole et al., 2020; Doetsch & Hen, 2005; Kerloch et al., 2019; Laplagne et al., 2006; Snyder, 2019; Stone et al., 2011; Tronel et al., 2015; Tuncdemir et al., 2019; Wu et al., 2015), with specific reference to plasticity heterogeneities. Further, these analyses will provide insights about the impact of neural heterogeneities, spanning adult‐ versus developmentally born mature as well as immature neurons, on location‐dependent plasticity profiles in the stratified synaptic inputs from lateral versus medial entorhinal cortices.

Second, as our focus here was on excitatory synaptic plasticity, we have not incorporated inhibition into our analyses. However, given the DG circuitry that recruits a diverse set of interneurons that impinge along different locations of the somato‐dendritic arbor (Amaral et al., 2007; Andersen et al., 2006; Dieni et al., 2013; Elgueta & Bartos, 2019; Freund & Buzsaki, 1996; Houser, 2007), it is essential that the impact of heterogeneities in inhibitory synaptic inputs on plasticity profiles are also assessed in more detail. In this context, there are lines of evidence that the inhibitory neurotransmitter GABA exerts functionally critical excitatory influences on the immature cells, and through the process of maturation shifts to being inhibitory (Chancey et al., 2013; Ge et al., 2006; Heigele et al., 2016). Thus, future studies that account for inhibition should also assess the impact of this switch in GABAergic impact on immature versus mature GCs and their plasticity profiles.

Third, whereas our *cellular‐scale* analysis has focused on the biophysical and structural heterogeneities as sources of plasticity heterogeneities, there are other potential sources for plasticity heterogeneities. At the molecular scale, it is possible that heterogeneities in the expression of plasticity related molecules (and associated signaling cascades) across synapses and across neurons of the same subtype could mediate plasticity heterogeneities (Josselyn & Frankland, 2018; Park et al., 2016). At the network scale, when multiple neurons are considered, pre‐existing afferent and local connectivity onto these neurons could form yet another potential source of plasticity heterogeneities (Josselyn & Frankland, 2018; Josselyn & Tonegawa, 2020). In addition, there are lines of evidence for a lower overlap in synaptic inputs impinging on immature GCs compared to inputs to mature GCs (Dieni et al., 2016), suggesting a role for afferent heterogeneities in not just regulating output correlations (Dieni et al., 2016; Mishra & Narayanan, 2019, 2021b) but also in mediating plasticity heterogeneities. Thus, future studies could expand the analyses of plasticity heterogeneity beyond the cellular scale to encompass network‐ and molecular‐scale components that could drive plasticity heterogeneities. In assessing plasticity heterogeneities at the network scale, it is important that the analyses are built on realistic networks of excitatory and inhibitory neurons receiving physiologically relevant local as well as afferent input activity. As the synapse‐localized calcium dynamics are critical mediators of synaptic plasticity, it is important that such analyses are performed on morphologically realistic neuronal models with realistic calcium dynamics and diffusion (Basak & Narayanan, 2018). At the molecular scale, performing realistic simulations would entail precise measurements of the different plasticity‐related signaling molecules in different synapses and assessing intra‐ and inter‐neuronal variability in the concentration of these signaling molecules across different synapses. Quantitative signaling cascades could then be built with realistic calcium inputs (Basak & Narayanan, 2018; Bhalla, 2004, 2014; Bhalla et al., 2002; Bhalla & Iyengar, 1999) to assess the molecular sources that mediate plasticity heterogeneity across GC synapses.

Fourth, our analyses here were limited to synaptic plasticity from the perspective of in vitro protocols. Of the two induction protocols employed in this study, although the 900 pulses protocol is an extremely useful biophysical tool to assess plasticity mechanisms (Anirudhan & Narayanan, 2015; Ashhad & Narayanan, 2013; Bienenstock et al., 1982; Castellani et al., 2001; Castellani et al., 2005; Cooper & Bear, 2012; Dudek & Bear, 1992; Honnuraiah & Narayanan, 2013; Narayanan & Johnston, 2010; Philpot et al., 2001; Shah et al., 2006; Shouval et al., 2002; Yeung et al., 2004; Yu et al., 2008), the physiological relevance is minimal given the requirement for 900 pulses for any given induction frequency. However, the TBS protocol carries considerable physiological relevance because the inputs from the lateral and the medial entorhinal cortices to the DG are theta modulated (Deshmukh et al., 2010). Therefore, the TBS protocol involves an activity pattern that is physiologically relevant in the context of the DG network (Beck et al., 2000; Bland, 1986; Buzsaki, 2002; Colgin, 2013, 2016; Davis et al., 2004; Diamantaki et al., 2016; Greenstein et al., 1988; Larson & Munkacsy, 2015; McHugh et al., 2007; Pavlides et al., 1988; Pernia‐Andrade & Jonas, 2014; Sainsbury & Bland, 1981; Shors & Dryver, 1994; Winson, 1974, 1978; Zhang et al., 2020).

In extrapolating our conclusions to an in vivo setting involving engram cell formation, it is essential that in vivo activity patterns and other forms of plasticity are considered as well. One route to approach plasticity in a network that incorporate different (intrinsic, synaptic, structural, and afferent) forms of heterogeneities studied here would be to use heterogeneous network models receiving activity patterns from the entorhinal cortices (Mishra & Narayanan, 2019, 2021b). Neuronal models and their connectivity should be constrained by the DG network, with plasticity implemented through the calcium control hypothesis employed here. In a heterogeneous conductance‐based setting, calcium through voltage‐ and ligand‐gated calcium channels could contribute to heterogeneous calcium influx across different neurons. The neuronal population could be constructed with mature or immature neurons, with differential connectivity and ion‐channel densities to provide insights about plasticity heterogeneities in an in vivo setting. Predictions from such heterogeneous biophysical networks could then be tested in DG networks using in vivo electrophysiology and/or population imaging of calcium activity in awake behaving animals.

Furthermore, plasticity in the DG GCs is known to span synaptic and intrinsic properties (Bliss & Lomo, 1973; Lopez‐Rojas et al., 2016; Mishra & Narayanan, 2021c, 2022). These observations necessitate future studies to assess the impact of neural heterogeneities on *conjunctive* intrinsic and synaptic plasticity, especially with reference to plasticity heterogeneities, resource allocation, and engram formation (Josselyn & Frankland, 2018; Josselyn & Tonegawa, 2020; Lisman et al., 2018; Mishra & Narayanan, 2021c; Park et al., 2016; Rao‐Ruiz et al., 2019; Silva et al., 2009). Analyses of the heterogeneities in such conjunctive plasticity involving multiple components, along with their roles in context‐specific resource allocation, could provide crucial insights about how the brain accomplishes stable and continual learning in an ever‐changing environment (Mishra & Narayanan, 2021c).

Finally, and importantly, our analyses emphasize the need to systematically characterize the expression of plasticity heterogeneities across different brain regions. Such analyses should span behavioral learning processes and pathological conditions to probe the mechanistic origins of and functional implications for plasticity heterogeneity. For instance, could pathology‐induced hyperplasticity that spans several neurological disorders (Bernier et al., 2011; Calabresi et al., 2003; Chattarji et al., 2015; Hulme et al., 2013; Kauer & Malenka, 2007; Markram & Markram, 2010; Rinaldi et al., 2008; Roozendaal et al., 2009; Soda et al., 2019) be a mechanism to reduce plasticity heterogeneity across neurons, thereby hampering context‐specific memory formation? Could loss of plasticity heterogeneity in the amygdala be a mechanism behind fear generalization that is observed with certain neurological disorders (Chattarji et al., 2015; Ghosh & Chattarji, 2015; Markram et al., 2008; Rahman et al., 2017; Suvrathan et al., 2014)? Could the ability of different neural‐circuit components—spanning transmembrane proteins, cytosolic and nuclear signaling elements, synaptic strength, neuronal morphology—to synergistically contribute to similar plasticity profiles (i.e., plasticity degeneracy) provide a therapeutic route for robustness in neural learning hampered by pathological conditions? These and other associated questions need to be systematically addressed through quantitative characterization of plasticity heterogeneities spanning different brain regions, physiological contexts, and pathological conditions, together assessing the implications for plasticity heterogeneities in context‐specific encoding of learned behavior.

## AUTHOR CONTRIBUTIONS

Sameera Shridhar, Poonam Mishra, and Rishikesh Narayanan designed experiments; Sameera Shridhar and Poonam Mishra performed experiments and carried out data analysis; Sameera Shridhar and Poonam Mishra, and Rishikesh Narayanan co‐wrote the paper.

## CONFLICT OF INTEREST

The authors declare that they have no competing interests.

## Data Availability

All data needed to evaluate the conclusions in the paper are present in the paper. This is a computational study, and no experimental data was generated as part of this study.
